# Competitive Interfacial Partitioning in Cetyltrimethylammonium Bromide Self‐Assembly: A Coarse‐Grained Study Across Deep Eutectic Solvents

**DOI:** 10.1002/open.70259

**Published:** 2026-07-21

**Authors:** Petteri A. Vainikka, Karen J. Edler

**Affiliations:** ^1^ Centre for Analysis and Synthesis, Kemicentrum Lund University Lund Sweden

**Keywords:** coarse‐grained molecular dynamics, cetyltrimethylammonium bromide (CTAB), deep eutectic solvents, Martini 3, surfactants

## Abstract

Understanding surfactant self‐assembly in deep eutectic solvents (DES) is fundamentally challenging, and computational efforts for in silico screening are frequently hampered by the computational expense of all‐atom simulations. To circumvent these limitations, we present newly parameterized and rigorously validated coarse‐grained Martini 3 models for the cetyltrimethylammonium (CTA) cation and glycerol. We deploy these models to unravel the anomalous phase behavior of cetyltrimethylammonium bromide (CTAB), which forms stable micelles in Glyceline (2:1 mixture of glycerol and choline chloride) but is macroscopically insoluble in the urea‐based DES, Reline (2:1 mixture of urea and choline chloride). By simulating surfactant titration across a mixed Glyceline‐to‐Reline gradient, we analyze solvent–component interactions with the micelles. Thermodynamic and spatial distribution analyses (MDDF, RDF, C(N), and SDF) reveal that this solvophobic effect is not electrostatically driven by the static choline cations. Rather, it is dictated by the competitive interfacial partitioning of the hydrogen‐bond donors. While dilute urea acts as an aggressive cosurfactant, increasing bulk urea concentrations trigger a thermodynamic bulk reclamation mechanism: the highly cohesive urea‐choline chloride network pulls urea away from the CTA headgroups, stripping the primary solvation shell and driving macroscopic precipitation. Ultimately, this demonstrates coarse‐grained molecular dynamics as a powerful, predictive in silico screening tool for solvent‐directed assembly.

## Graphical abstract

Martini coarse‐grained simulations probe solvent–surfactant interactions of cetyltrimethylammonium bromide in choline chloride‐based deep eutectic solvents containing glycerol or urea. Competitive partitioning of the hydrogen‐bond donors around the surfactant headgroups accompanies striking composition‐dependent changes in micellar self‐assembly: glycerol‐rich mixtures retain near‐globular aggregates, whereas urea‐rich mixtures form elongated aggregates at elevated temperature.

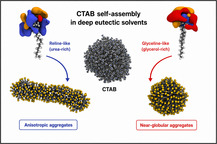

## 1 Introduction

Deep eutectic solvents (DESs) are mixtures of hydrogen bond donors and acceptors [1], which have shown promise in gas sequestration [2], energy storage [3, 4], specific extraction [5], and solubilization of otherwise poorly soluble pharmacologically active components [6]. DESs share many of the same properties as ionic liquids: low vapor pressure, low flammability, and high tunability [7]. Furthermore, DESs are often reasonably cheap to produce [8]. With care in choosing the precursors, DESs can be made from environmentally benign components, and as the purity of the formed DES is directly linked to the purity of the precursors, they can be easily manufactured in a pure state [8].

In the past decade, DESs have been studied and utilized as a medium for surfactant self‐assembly [9, 10, 11]. For instance, Arnold et al. [12] reported that sodium dodecyl sulfate (SDS) forms elongated micelles when placed in Reline (urea:choline chloride DES, formed in a 2:1 ratio), while Sanchez‐Fernandez et al. [13] reported globular SDS micelles in Glyceline (glycerol:choline chloride, formed in a 2:1 ratio). These studies often involve small‐angle neutron scattering (SANS) and small‐angle X‐ray scattering methods, which yield information about the global shapes of the micelles present (e.g., micellar aspect ratio), but yield relatively little information regarding the molecular‐ or atomic‐level interactions that drive the self‐assembly and solvation of surfactants.

Computational tools have been applied for these studies regularly [14, 15]. All‐atom (AA) molecular dynamics (MD) simulations are routinely used to study kinetic, structural, and thermodynamic properties of surfactant‐bearing DES systems [16, 17]. Although the atomistic detail provides excellent accuracy, it is computationally resource‐intensive, prohibiting the simulations of large systems (>10^5^ atoms) over longer timescales (>1 µs). In the specific case of surfactants, this poses a major challenge: in order to simulate systems near the critical micelle concentration (CMC) in such a way that the simulated system contains a meaningful amount of surfactant molecules (>10^2^), one has to simulate a massive amount of solvent. This often results in the need to utilize HPC resources, which are not universally available. Moreover, at times, the exact equilibrium structure of a micelle is not known, precluding the use of a preordained near‐equilibrium starting structure. This leads to a dilemma: either one starts from a random, unbiased surfactant configuration, which leads to massive resource challenges in terms of simulation length, as self‐assembly is a diffusion‐limited process and can take hundreds of nanoseconds, or one starts from a completely biased starting configuration, potentially leading to sampling from a kinetically trapped, incorrect state.

One way to circumvent these challenges is to reduce the degrees of freedom of the models used by coarse‐graining (CG) the resolution. Multiple CG force fields contain parameters for common surfactants (e.g. SDS, cetyltrimethylammonium bromide (CTAB)), monovalent ions, and water. However, to the authors’ knowledge, there is currently only one top‐down CG force field with transferable parameters necessary for simulations of surfactants in DES:MARTINI 3 [18]. The authors’ previous work has established the accuracy of the DES models themselves [19, 20], and recent work has demonstrated the efficacy of this approach in simulating DES‐surfactant systems [21]. This approach not only allows for the verification and refinement of experimental results but also enables the possibility of screening surfactant phase behavior and dynamics in DESs. This approach is further enhanced by the readily available tools for backmapping simulations, enabling the use of CG MD as an equilibration step for self‐assembly, and allowing AA‐level details, which might be required for, for example, SANS curve prediction.

Taken together, the approach presented here is part of an ongoing effort to move classical computational tools beyond post hoc analysis toward the role of in silico screening tools for surfactant self‐assembly in DESs. Here, we apply this approach to the unresolved phase behavior of the cationic surfactant cetyltrimethylammonium bromide (CTAB). While anionic surfactants such as SDS adopt strongly solvent‐dependent aggregate morphologies in Glyceline and Reline, CTAB forms stable globular micelles in glycerol‐based Glyceline but is macroscopically insoluble in pure Reline. The molecular‐level basis of this contrast remains unresolved. Because Glyceline and Reline differ principally in their hydrogen‐bond donors, glycerol and urea, respectively, a central question is how these components partition at the cationic micelle‐solvent interface.

To address this question, this work first details the parameterization of novel Martini 3 models for glycerol and the CTAB surfactant. We demonstrate that these models are transferable, successfully reproducing known macroscopic mesophases and thermodynamic properties. Finally, applying these validated models to a mixed Reline‐Glyceline solvent gradient, we characterize composition‐dependent CTAB morphology and CTA‐solvent interfacial partitioning. This provides a molecular‐level description of solvent distributions associated with the unusual phase behavior of CTAB.

## 2 Results and Discussion

### 2.1 Coarse‐Grained Model Parameterization and Validation

To accurately investigate the solvent‐directed assembly of CTAB in DESs, novel coarse‐grained models for the cetyltrimethylammonium cation (CTA) and glycerol (GLYC) were parameterized for the Martini 3 force field. A full description of the parameterization scheme is given elsewhere [18, 22], and only the key values will be discussed here. All parameterization details are given in full in the Supporting Information, section 1.

As no well‐defined log *p* value can be extracted for the CTA cation, the correctness of the mapping was validated by comparing the experimental and predicted free energies of micellization in water, which differed by 1.77 kJ/mol [23]. This is in excellent agreement with experimental estimates, with the observed deviation comparable to ambient thermal fluctuations.

Similarly, the uncharged glycerol model was parameterized and validated against partitioning and volumetric data. The Martini 3 glycerol model yielded a Log P of −2.00, closely aligning with the predicted (−1.93) and experimental (−1.76) values. Furthermore, the temperature‐dependent density of pure glycerol was evaluated against experimental data. The linear function for the Martini 3 density exhibited a maximum deviation from the experiment of 7% across the entire tested temperature range, with an average deviation of just 5%. The discrepancy decreases with increasing temperature and is approximately 2.6% at 350 K.

Finally, to establish that the newly derived CTA cation model is fully transferable and capable of replicating complex self‐assembly, the model was tested for its ability to reproduce known macroscopic mesophases. The coarse‐grained surfactant successfully assembled into both a structurally stable hexagonal phase and a bicontinuous sponge phase in water [24]. (see Supporting Information, Section 1.1.1 for structural details) Having established the thermodynamic and structural accuracy of these foundational models, we subsequently deployed them to investigate the anomalous insolubility of CTAB across a Reline‐to‐Glyceline gradient.

### 2.2 Micelle Morphology and Self‐Assembly

**FIGURE 1:**
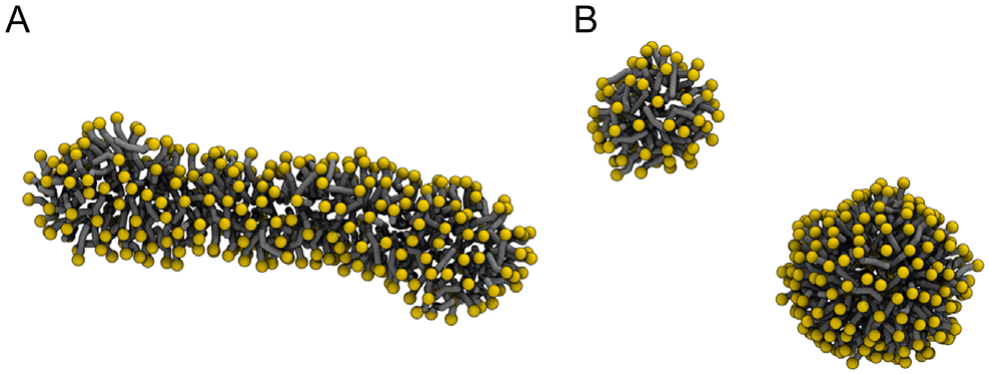
SDS micelle morphologies in (A) Reline and (B) Glyceline. The wormlike micelle quickly breaks to form two globular micelles.

We began this section of the study with a control simulation: we have established earlier that our DES and SDS surfactant models are suitable for studying self‐assembly and morphological changes [21]. With a working glycerol model, we could now test if we could recreate the findings of Sanchez‐Fernandez et al. [13], who reported globular SDS micelles in Glyceline. We first allowed SDS to form a stable, highly elongated worm‐like micelle in pure Reline, before solvating it in pure Glyceline. Upon equilibration, the elongated structure rapidly collapsed, undergoing fission to form two distinct globular micelles. This observation, illustrated in Figure 1, confirms that the distinct hydrogen‐bond donor networks in these two DESs actively dictate the aggregate's preferred surface‐to‐volume ratio.

In contrast to SDS, CTAB is experimentally insoluble in pure Reline [25]. To examine the effect of solvent composition on CTAB aggregate morphology and CTA interfacial solvation, we simulated CTAB aggregates across a mixed Glyceline‐to‐Reline composition gradient. Aggregate morphology was quantified by extracting the principal radii ($R_{1}$, $R_{2}$, and $R_{3}$), the total radius of gyration ($R_{g}$), and the relative shape anisotropy ($K^{2}$) from the mass‐weighted gyration tensor. The corresponding morphological data are given in full in the Supporting Information, Tables S7 and S8.

At 350 K, CTAB aggregate morphology is composition‐dependent. In the glycerol‐rich 90:10 Glyceline:Reline mixture, the aggregate remains near‐globular, whereas the 50:50 and 30:70 mixtures form distinctly anisotropic, nonspherical aggregates. These elevated‐temperature simulations demonstrate a composition‐dependent departure from near‐globular CTAB aggregation in urea‐rich mixtures, similar to that observed experimentally [25]. Detailed morphological parameters are given in the Supporting Information, Table S8; representative snapshots and corresponding radial distribution functions (RDFs) are provided in Figures S8 and S10, respectively.

At 298 K, the CTAB aggregates retain low anisotropy across the full solvent gradient, with mean $K^{2}$ values below 0.01 and block‐bootstrap 95% confidence intervals remaining below this threshold. However, this temperature lies below the experimentally reported Krafft temperature of CTAB in these mixtures [25]. The low‐temperature aggregate morphologies are therefore treated as kinetically constrained reference states on the simulated timescale, rather than equilibrium aggregate shapes or direct predictions of macroscopic CTAB solubility. These persistent near‐globular aggregates provide morphology‐constrained reference states for the composition‐resolved interfacial analysis below. In contrast, SDS elongates at 298 K in Reline, consistent with its lower Krafft temperature in these DES mixtures [25].

### 2.3 Interfacial Solvation of CTAB

**FIGURE 2:**
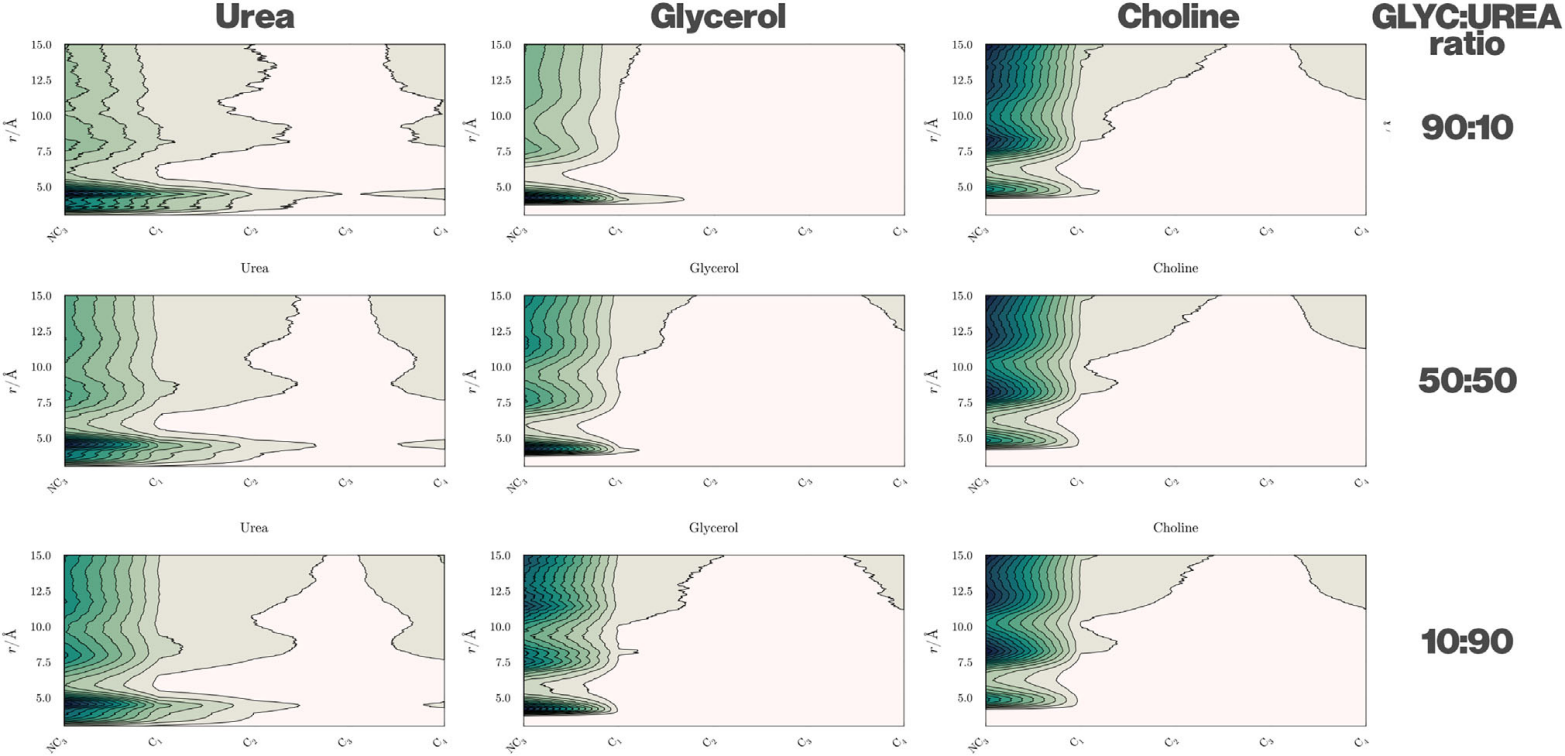
2D Minimum Distance Distribution Function (MDDF) contour maps mapping the spatial distribution of urea, glycerol, and choline relative to the CTA surfactant backbone at 90:10, 50:50, and 10:90 Glyceline:Reline ratios. The density profiles highlight the pronounced interfacial localization of urea at low bulk concentrations and the corresponding physical exclusion of glycerol.

Using the persistent near‐globular CTAB aggregates at 298 K as morphologically constrained reference states, we next examine the molecular‐level composition of the CTA‐solvent interface. This analysis compares composition‐dependent solvent partitioning around a common low‐temperature aggregate morphology. To quantify the spatial distribution and competitive partitioning of the solvent components along the surfactant backbone, 2D Minimum Distance Distribution Functions (MDDFs) and corresponding density maps were generated. Key findings from this analysis are given in Figure 2, and the full data are available in the Supporting Information, Section 4.

The MDDF contour plots reveal three distinct solvophobic behaviors that drive the interfacial thermodynamics. The spatial distribution of the choline cation remains completely static regardless of the solvent ratio. Due to electrostatic repulsion with the CTA headgroups, choline is localized to the secondary solvation shell. The invariant distribution indicates that the bulk cationic environment of the DES does not actively drive the interfacial structural changes.

In contrast, the hydrogen‐bond donors exhibit nonideal, competitive partitioning. Notably, urea demonstrates its maximum interfacial localization at its lowest bulk concentration. In the glycerol‐rich 90:10 Glyceline:Reline system, urea partitions strongly to the polarized surfactant headgroups, localizing at the interface to effectively act as a cosurfactant despite its minority status in the bulk solvent.

This localized partitioning of urea corresponds to a pronounced exclusion of glycerol. The MDDF plots reveal a distinct glycerol depletion zone at the micellar interface. Counterintuitively, this depletion zone becomes stronger and more pronounced as the bulk concentration of glycerol increases. In the 90:10 mixture, the abundant bulk glycerol is effectively displaced from the primary interfacial binding pockets by the highly localized urea.

Taken together, these profiles demonstrate that the interfacial environment is not governed by mere electrostatics, but rather by the concentration‐dependent, competitive partitioning of the hydrogen‐bond donors at the micelle surface.

### 2.4 Quantitative Solvation Shell Composition

**FIGURE 3:**
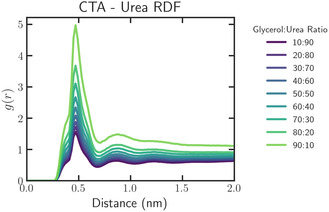
Urea‐CTA RDF. The primary solvation peak at 0.45 nm demonstrates a severe inverse concentration dependence, reaching its maximum intensity (g(r)≈5.0) at the lowest bulk urea concentration (90:10 Glyceline:Reline).

RDFs were computed for the solvent components relative to the CTA headgroups and are presented in the Supporting Information, Section 3. The resulting profiles physically corroborate the divergent partitioning mechanisms observed in the 2D density maps. The CTA choline RDF remains structurally invariant across the entire titration gradient, with its primary peak relegated to a distance of r≈0.85 nm. The CTA‐glycerol RDF scales predictably, with its primary peak intensity (r≈0.45 nm) scaling in direct proportion to its bulk concentration. However, the CTA‐urea RDF reveals a strong inverse concentration dependence, shown in Figure 3.

In the highly dilute 10% Reline system, urea exhibits a sharp localization peak (g(r)≈5.0) at r≈0.45 nm. As the bulk fraction of Reline increases towards 90%, the primary peak decreases by more than a factor of three (g(r)≈1.5). This pronounced inverse concentration dependence demonstrates that urea becomes progressively less preferentially enriched at the CTA interface as its bulk concentration increases. The RDFs therefore show that the interfacial distribution of urea is not governed by bulk availability alone, but instead changes substantially across the Glyceline‐Reline composition gradient.

**FIGURE 4:**
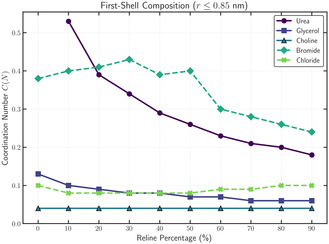
First‐shell coordination numbers (C(N)) for urea, glycerol, choline, and the counterions (bromide and chloride) as a function of bulk Reline percentage.

To provide further quantitative thermodynamic basis for the partitioning phenomena observed in the MDDFs, the absolute composition of the primary solvation shell (r≤0.85 nm) was evaluated by extracting the coordination numbers (C(N)) for each solvent component across the entire titration range, as is shown in Figure 4.

Data in Figure 4 further confirms the qualitative observations derived from the 2D density maps. The C(N) values for both the choline cation and the chloride anion remain consistently flat across all solvent ratios. Choline maintains a near‐zero presence in the primary shell (C(N)≈0.04), indicating secondary‐shell localization, as also observed in the MDDFs. The comparatively invariant distributions of these ionic components indicate that they do not govern the observed composition dependence of the CTA interfacial environment.

Conversely, the neutral hydrogen‐bond donors exhibit strongly composition‐dependent interfacial populations. Urea reaches its maximum first‐shell coordination at 10% Reline, with C(N)≈0.53. As the bulk urea concentration increases towards the Reline‐rich end of the composition gradient, its absolute first‐shell population decreases by approximately a factor of three, reaching C(N)≈0.18 at 90% Reline.

This inverse concentration dependence shows that the interfacial distribution of urea is not governed by bulk availability alone. In particular, the observed decrease in absolute urea coordination is inconsistent with a simple saturation of fixed high‐affinity interfacial sites, for which the first‐shell population would instead be expected to increase or approach a plateau as the bulk urea concentration rises. Rather, urea becomes progressively less preferentially associated with the CTA interface in Reline‐rich mixtures. The reduced interfacial urea population is qualitatively consistent with experimental SANS modeling, which indicated little or no solvent contribution in the CTAB headgroup region at high urea contents in choline chloride:urea:glycerol mixtures [25].

### 2.5 Spatial Density Functions and Solvation Shell Morphology

**FIGURE 5:**
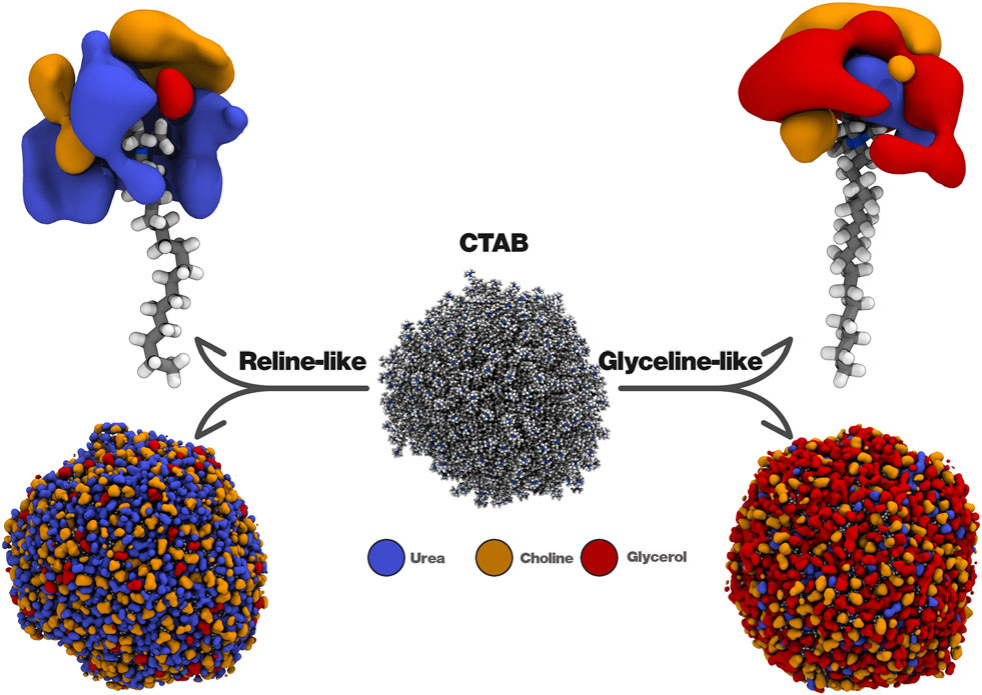
SDFs illustrating the localization of urea (blue), choline (orange), and glycerol (red) around the CTA micelle and surfactant. The top row depicts the local binding pockets on a single extracted CTA molecule, while the bottom row displays the general solvation scheme of a CTAB micelle in Reline‐like (left) and Glyceline‐like (right) solvent environments.

As a final effort to visualize the 1D and 2D data shown above, Spatial Density Functions (SDFs) were rendered and are shown in Figure 5. These density‐based volumetric maps illustrate the high‐probability binding pockets around both a single CTA cation and the fully assembled micelle in Reline‐like high‐urea‐content and Glyceline‐like high‐glycerol‐content environments.

In the Glyceline‐like environment (Figure 5, right), the general micellar surface is overwhelmingly solvated by the abundant glycerol (red). However, the single‐molecule SDF reveals a high‐probability urea density directly adjacent to the CTA headgroup. Together with the MDDF, RDF, and coordination number analyses, this indicates preferential enrichment of urea at the CTA interface in glycerol‐rich mixtures, accompanied by depletion of glycerol from the same interfacial binding pockets.

Conversely, in the Reline‐like environment (Figure 5, left), the aggregate is surrounded primarily by urea and choline. Although urea remains the dominant interfacial species by volume, the SDF lobes around a single CTA molecule are more diffuse and bulk‐oriented than the sharp, localized urea pocket observed in the Glyceline‐rich system. These density maps therefore provide a three‐dimensional representation of the composition‐dependent shift from localized urea enrichment at low bulk urea concentration to a broader and less CTA‐proximal urea distribution in Reline‐rich mixtures.

The choline cation remains localized primarily in the outer, secondary solvation shell in both environments, distinctly separated from the CTA headgroup. These three‐dimensional visualizations are consistent with the trends observed in the one‐ and two‐dimensional analyses and further illustrate the composition‐dependent redistribution of the hydrogen‐bond donors at the micelle‐solvent interface.

## 3 Conclusion

In this work, we investigated the structural and interfacial behavior of the cationic surfactant CTAB across a Glyceline‐Reline solvent gradient. To enable these simulations, we parameterized and validated Martini 3 coarse‐grained models for the CTA cation and glycerol against atomistic, thermodynamic, and mesophase benchmarks. The resulting models reproduce experimentally relevant surfactant self‐assembly behavior and provide a computationally efficient framework for studying DES‐directed assembly.

Comparison with SDS demonstrates that the response to changing DES composition depends strongly on surfactant identity. At 350 K, CTAB aggregate morphology becomes composition‐dependent. In the glycerol‐rich 90:10 Glyceline:Reline mixture, the aggregate remains near‐globular, whereas the 50:50 and 30:70 mixtures form anisotropic nonspherical aggregates. In contrast, at 298 K, CTAB retains low anisotropy across the full Glyceline‐Reline gradient. Because this temperature lies below the experimentally reported Krafft temperature of CTAB in these mixtures, the low‐temperature aggregates are treated as morphologically constrained reference states rather than equilibrium aggregate shapes.

The 298 K interfacial analyses reveal that the composition‐dependent CTA solvation environment is not governed by the choline cations, whose distribution remains largely invariant and confined to the secondary solvation shell. Instead, the CTA interface is characterized by competitive partitioning of the hydrogen‐bond donors. In glycerol‐rich mixtures, urea displays pronounced preferential enrichment adjacent to the CTA headgroups despite its low bulk concentration, accompanied by local glycerol depletion. As the bulk urea fraction increases, however, the absolute first‐shell urea population decreases substantially. This inverse concentration dependence is inconsistent with a simple saturation of fixed interfacial binding sites and demonstrates that urea becomes progressively less preferentially associated with the CTA interface in Reline‐rich mixtures.

These results provide a molecular‐level description of the solvent‐dependent interfacial environment of CTAB and are qualitatively consistent with SANS observations of reduced solvent contribution in the CTAB headgroup region at high urea content. More broadly, they demonstrate that coarse‐grained MD can be used to identify composition‐dependent interfacial partitioning and morphology in surfactant‐containing DES systems, supporting its use as an in silico screening approach for solvent‐directed self‐assembly.

## 4 Computational Methods

### 4.1 Computational Details

All simulations were performed with GROMACS 2024.2 [26, 27]. Novel models created for CTAB and glycerol were tested with GROMACS 2021.7 in order to ensure compatibility across different versions of the simulation software. All CG simulations were run with a 20 fs timestep. All AA simulations used a 1 fs timestep. Pressure was controlled with the stochastic cell rescaling algorithm [28]. Pressure was set to 1 bar, and the compressibility was set to 3e−4 $bar^{-1}$ for CG simulations and 4.5e−5 $bar^{-1}$ for AA simulations. Temperature was controlled with the Bussi‐Donadio‐Parrinello thermostat (V‐rescale) [29] and was set to 298 K unless otherwise stated. The elevated‐temperature CTAB morphology simulations were conducted at 350 K. Constraints used for H bonds in AA simulations and for general bonded terms in the CG simulations were solved using LINCS [30] with a LINCS order of 8. Electrostatic interactions were computed using the smooth particle mesh Ewald (PME) method for both CG and AA simulations. Backmapping from Martini 3.0 to CHARMM36 [31] was performed using CG2AT [32]. Thermodynamic Integration (TI) routines employed 21 λ‐points with soft‐core potentials applied as sc−α = 0.5 and sc−p = 1. Results were compiled using Bennett's acceptance ratio. MDDFs and MDDF‐based density plots were derived using the ComplexMixtures [33] Julia package. Radial distribution functions (RDFs) and coordination numbers (CNs) were derived using the Freud Python package [34]. Simulation volume unwrapping, manipulation, and micelle morphological parameters were derived using MDAnalysis [35]. Micelle morphological parameters, including the principal radii and the relative shape anisotropy $\left(K^{2}\right)$, were extracted by computing the eigenvalues of the mass‐weighted gyration tensor for the largest continuous surfactant cluster in each frame. Spatial density functions (SDFs) were obtained using the *VolMap* plugin, distributed as part of the VMD software [36].

### 4.2 CG Models

**FIGURE 6:**
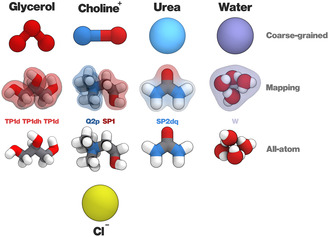
The main solvent components used in this study. Note that water at the Martini resolution represents four water molecules. Ions are represented by individual beads and follow a 1:1 mapping. The beads are not drawn to scale.

**FIGURE 7:**
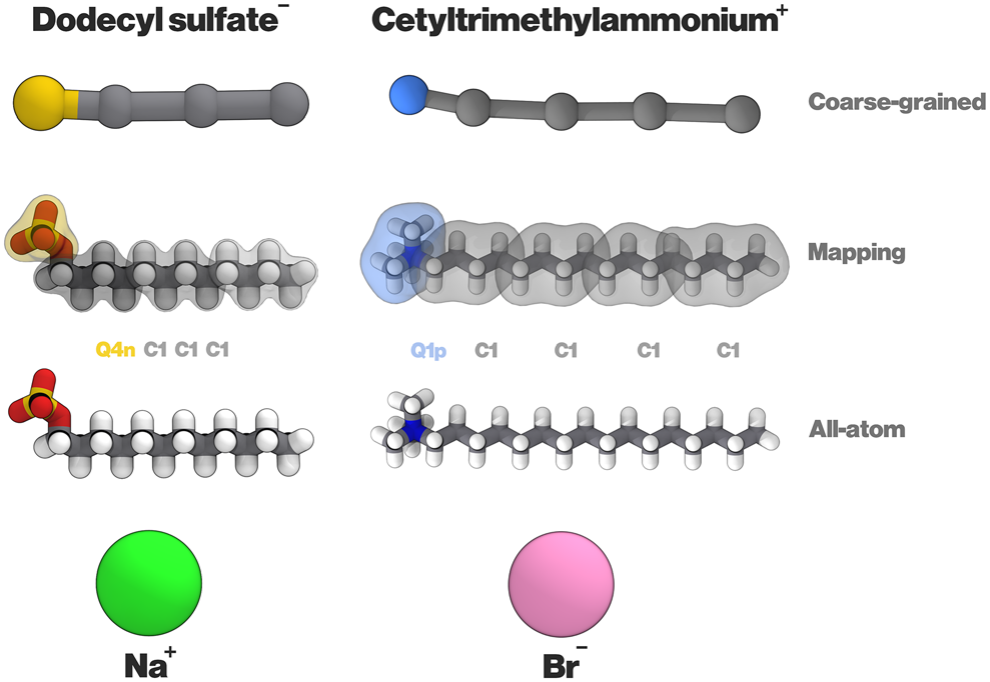
Surfactants used in this work.

This work utilized the main author's previously published models on DESs [19, 20] for dodecyl sulfate, urea, and choline. Martini 3 models for glycerol and cetyltrimethylammonium cation were parameterized for this work (see SI Section 1 and the following subsections for more details). Models for water and the counterions (bromide and chloride) were obtained from the published Martini 3.0 library, which is distributed freely with the force field [18]. Solvent models used in this work are given in Figure 6, and the surfactant models with corresponding counterions are presented in Figure 7.

### 4.3 Simulation Setups

CG simulation setups all followed the following scheme: Initial configurations were obtained by randomly placing components into the simulation volume using the built‐in GROMACS tool *gmx insert molecules*. All systems were then subjected to a steepest‐descent energy minimization routine, followed by equilibration steps in the isobaric‐isothermal ensemble: First, a 100 ps simulation at 200 K with a 1 fs timestep, followed by a 500 ps simulation at 290 K using a 2 fs timestep. This was followed by a 2 ns simulation at the same temperature, using a 10 fs timestep. The final equilibration step used a 20 fs timestep, lasting for 50 ns at 290 K. All production simulations containing surfactants were run for 5 µs, with the exception of the elevated temperature simulations, which were run for 500 ns.

This work employed AA simulations for two specific tasks. First, they were used to derive bonded terms for the novel Martini models created for this work. Second, backmapped AA simulations were used to derive both micelle‐ and surfactant‐level SDFs. The former simulations involved placing a single copy of either CTAB or glycerol in a 4×4×4 nm volume and solvating it with TIP3P water. These systems were then minimized using the steepest‐descent algorithm, equilibrated using a 1 fs timestep for 500 ps in the canonical ensemble, followed by a 1 ns equilibration with a 2 fs timestep in the isothermal–isobaric ensemble with 1 bar and 298 K. Finally, a production run using the same settings from the previous equilibration step was performed, lasting for 150 ns. For the latter case of simulating backmapped systems, we employed the same minimization and equilibration routine. The production simulations had the same settings as well, but lasted for 300 ns.

## Funding

This study was supported by the Wenner‐Gren Foundation (UPD2023‐0035).

## Conflicts of Interest

The authors declare no conflicts of interest.

## Supporting information

Supplementary Material

## Data Availability

The data that support the findings of this study are available in the Supporting Information of this article, and the coarse‐grained models and structures parameterized in this study are available on GitHub: https://github.com/vainikanpete/martini3‐DES‐models.
