# Supplementary material for: Competitive Interfacial Partitioning in Cetyltrimethylammonium Bromide Self‐Assembly: A Coarse‐Grained Study Across Deep Eutectic Solvents
Source: ChemistryOpen. 2026 Jul 21;15(8):e70259. doi: 10.1002/open.70259 (PMC13388030; doi:10.1002/open.70259)
Supplement: Supplementary file 1 — Supplementary Material [file OPEN-15-e70259-s001.pdf]

Competitive Interfacial Partitioning in CTAB Self-Assembly: A Coarse-Grained Study Across Deep Eutectic Solvents

Supporting Information

Petteri A. Vainikka[1]*, Karen J. Edler[1]*

[1] Centre for Analysis and Synthesis, Department of Chemistry, Lund University, Box 124, 221 00 Lund, Sweden

* Corresponding Author
Email: petteri.vainikka@chem.lu.se

Number of pages: 24
Number of figures: 20
Number of tables: 8

1. Coarse-grained models parameterized for this study

The following subsections introduce both new compounds. The sections contain the mapping scheme, list of validated properties, full CG topology, and some rationales behind the parameterization choices.

1.1. Cetyltrimethylammonium-cation (CTA)

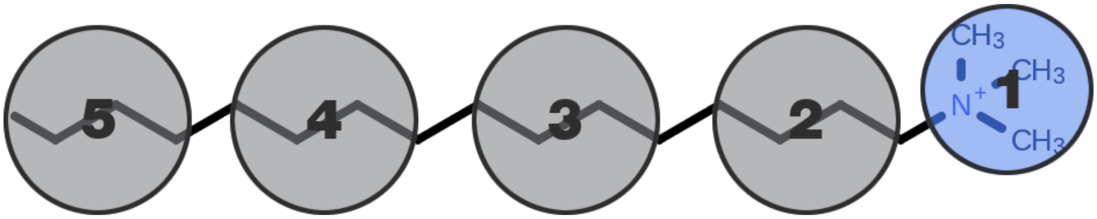

Figure S1: Mapping scheme for the CTA-cation. Numbers on the beads correspond to Table S1.

Table S1: Topology of Martini 3 CTA-cation.

Bead definitions			
**Number**	**Bead type**	**Charge**	**Mass**
1	SQ2p	1.00	59
**2**	C1	0.00	56
**3**	C1	0.00	56
**4**	C1	0.00	56
**5**	C1	0.00	57

Bonds				
i	***j***	**Function**	**Length**	**Force c.**
**2**	3	1	0.45	5000
**3**	4	1	0.45	5000
**4**	5	1	0.475	5000

Constraints			
i	***j***	**Function**	**Length**
**1**	2	1	0.365

Angles					
i	*j*	*k*	Function	Angle	Force c.
**1**	2	3	2	150	20
**2**	3	4	2	140	15
**3**	4	5	2	140	15

Ryckaert-Bellemans Dihedrals										
i	*j*	*k*	*l*	Function	C0	C1	C2	C3	C4	C5
**1**	2	3	4	3	1.4	0.2	-0.1	0.15	-0.15	-0.1
**2**	3	4	5	3	1.4	0.2	-0.1	0.15	-0.15	-0.1

As CTA-cation acts as a surfactant, there is no reliable partitioning data available. In lieu of this, we measured the ΔG of micellization by establishing a TI cycle (see *'Computational Details'* section of the main text for further details) for a free surfactant in water to a surfactant assembled in micelle. This data could then be related to that derived from microcalorimetric studies. The difference between our models prediction and experiment is less than that ambient of thermal noise.

Table S2: Measured thermodynamic properties for CTA-cation.

Property	Martini 3	Experimental	Ref.
**ΔG of micellization**	-35.77 ± 0.07 kJ /mol	-34.0 kJ/mol	1

Table S3: Solvent accessible surface area (SASA). AA value is derived using Rowland's atomic radii. Positive difference indicates the Martini model to be larger than the atomistic model, and vice versa.

Martini 3 (nm^2)	All atom (nm^2)	Difference (%)
7.406 ± 0.29	7.676 ± 0.19	-3.52

1.1.1 Cetyltrimethylammonium-cation surfactant properties

In order to establish that our CTA-cation model is transferable and capable of producing high-quality data, we reproduced two known mesophases of CTAB: A hexagonal phase and a bicontinuous sponge phase. The former is shown in Figure S2 below.

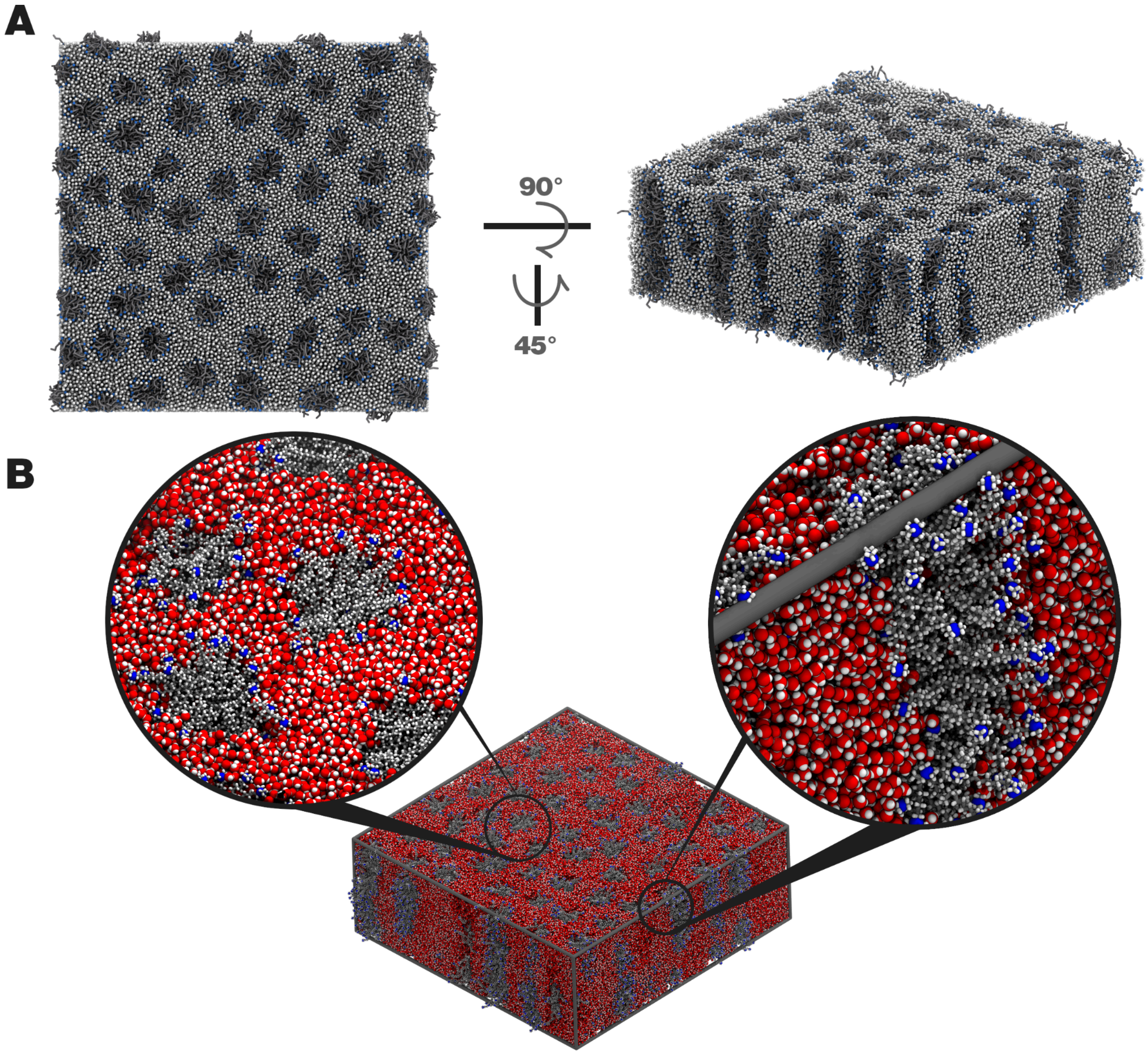

Figure S2: CTAB hexagonal phase. A) CG system after 750 ns of simulation, shown from the top and side. The system is 40 x 40 x 15 nm³ in volume, containing 233,600 beads. Water beads are drawn in white, the aliphatic tails of CTAB in gray and the head groups in blue. B) The same system, backmapped to 2,662,800 atoms. Water is now in red and white, CTA-cations follow the colour scheme from earlier.

The structural classification of the hexagonal phase was determined by evaluating the positional ratios of the coordination peaks in a 2D RDF, shown in Figure S3 below.

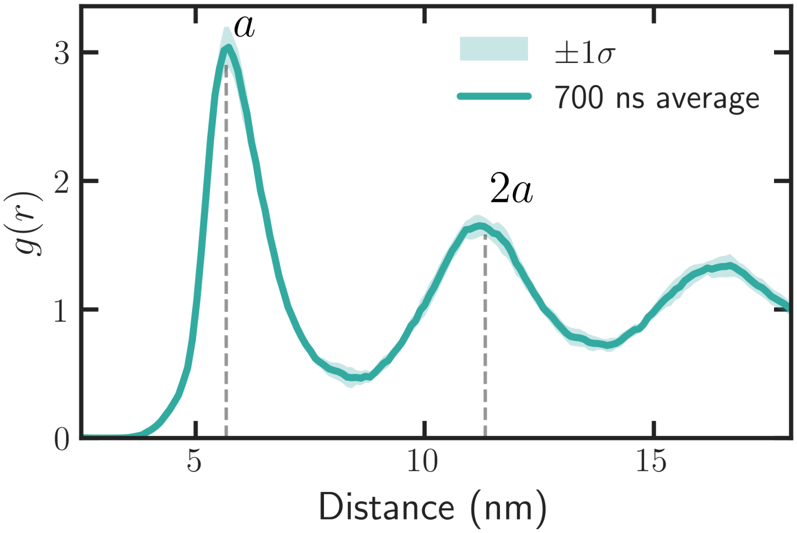

Figure S3: 2D RDF of the CTAB hexagonal phase. Primary peak is annotated as ‘a’, secondary as ‘2a’. Light shaded region indicates standard deviation.

The primary peak, ‘a’, denotes the nearest-neighbor inter-cylinder distance, representing the lattice parameter of the hexagonal ensemble. The presence of a distinct secondary peak at ‘2a’, along with the characteristic smearing of the $\sqrt{3}$a coordinate into its leading edge, confirms the long-range positional order inherent to the lyotropic hexagonal phase. The data is averaged over 700 ns of trajectory and the dynamic nature of the system (i.e., the thermal fluctuations of the cylinder centers of mass) naturally broadens the RDF peaks. In order to establish that the system is indeed in an equilibrium, a block analysis was performed on 100 ns blocks over the whole trajectory. This is shown in Figure S4 below.

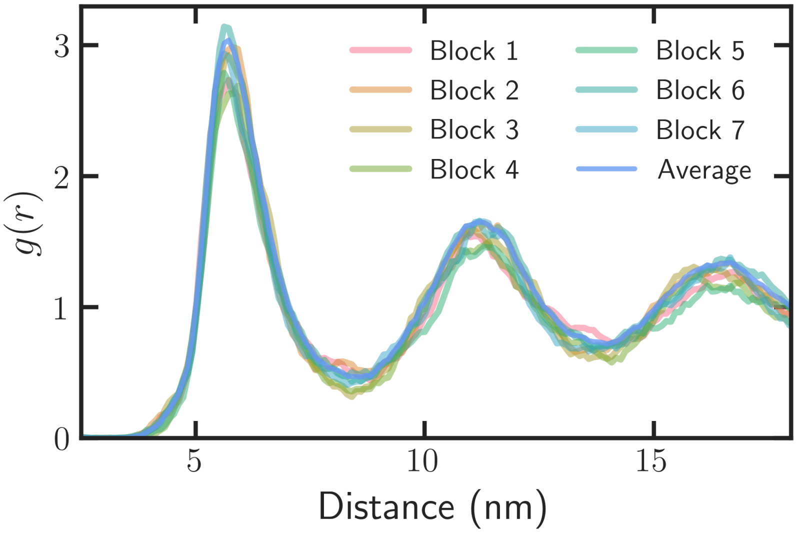

Figure S4: Block analysis of the cylinder center-of-mass 2D RDF calculated over 100 ns blocks. The consistent peak alignment across all intervals demonstrates that the hexagonal phase is fully equilibrated, with variations strictly limited to expected thermal noise.

Defining the presence of a bicontinuous sponge phase was performed in a two-pronged approach: Firstly we performed a percolation analysis on the system shown in Figure S5, by running two parallel clustering analyses: one on the hydrophobic tail of the CTA-cation, the other on the water beads.

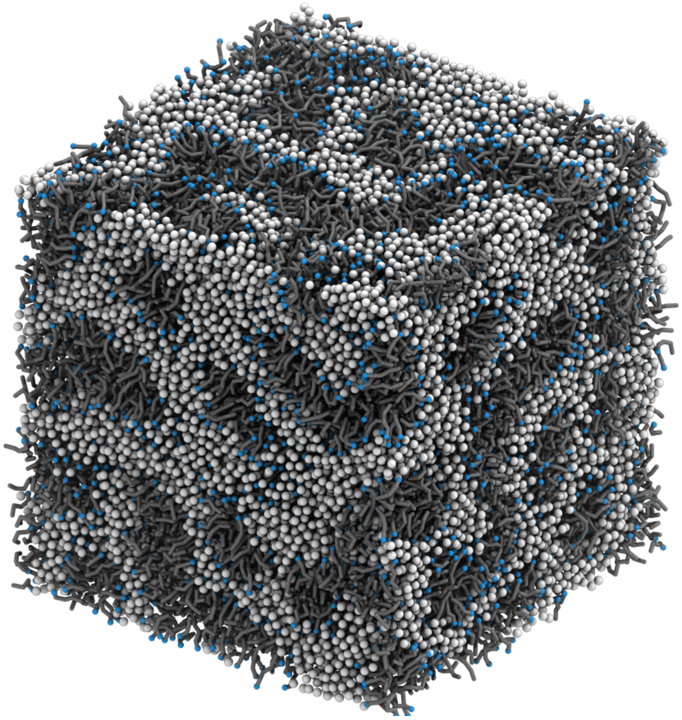

Figure S5: A bicontinuous phase with water and CTAB. Colour scheme follows earlier figures.

Distance-based clustering revealed that 99.9% of the polar solvent molecules formed a single, unbroken 3D network spanning the simulation box. Simultaneously, 94.0% of the hydrophobic surfactant tails were interconnected within a single macro-cluster. The simultaneous crossing of the percolation threshold (set at 90%) by both the polar and apolar domains definitively classifies the system as a true dual-continuous phase, ruling out the presence of dynamically arrested, isolated micellar aggregates.

Finally, to quantify the geometry of the bicontinuous mesophase, the Teubner-Strey macroscopic continuum model was fitted to the RDF of the surfactant tail beads:

$$g(r) = 1 + \frac{A}{r}e^{-r/\xi} \times sin(2\pi r/d + \phi)$$

where d is the characteristic domain spacing, ξ is the correlation length and A and ϕ are the amplitude and phase shift, respectively.

To isolate the macroscopic inter-domain oscillations from local microscopic bead-bead packing, the fitting window was strictly constrained between 3.5 nm and 10.0 nm. Fits were optimized using the Levenberg-Marquardt algorithm and goodness-of-fit was evaluated using the coefficient of determination and root-mean-square error, both of which are reported alongside Figure S6 below, showing the fit.

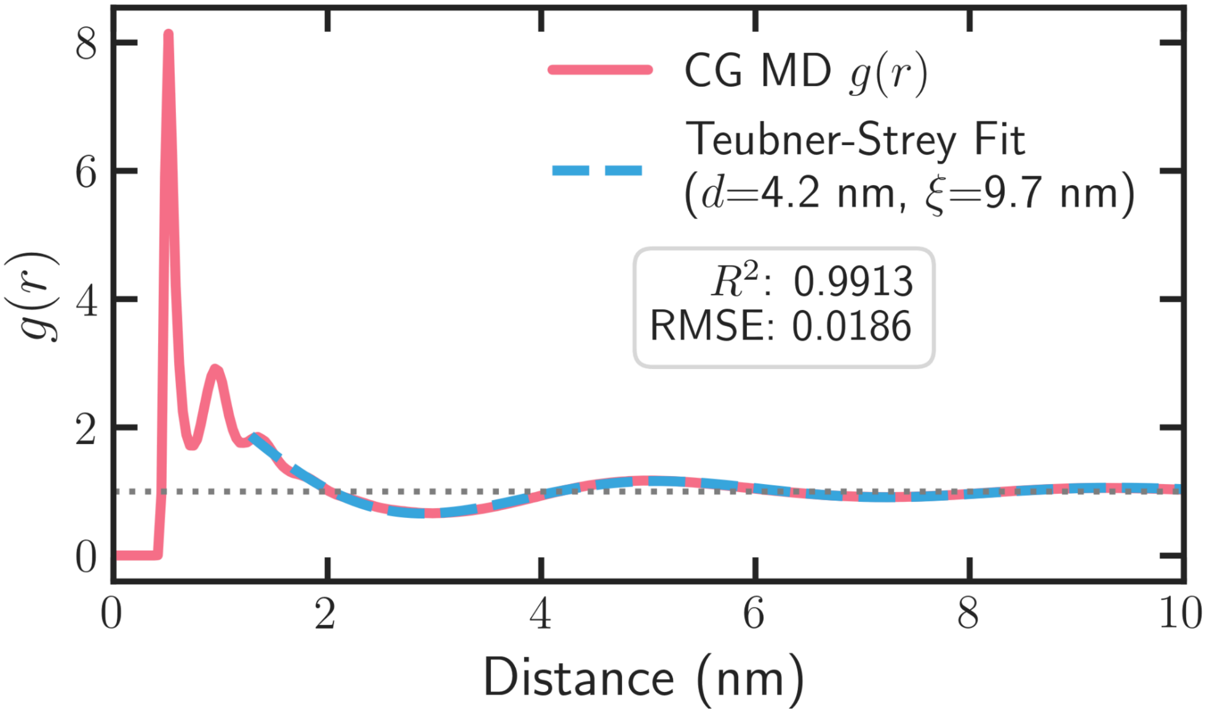

Figure S6: RDF of the CTA⁺ tail beads in the bicontinuous sponge phase. The Teubner-Strey model (dashed blue line) is fitted to the coarse-grained MD data (solid pink line) over a constrained fitting window of 3.5 to 10.0 nm, isolating large-scale inter-domain oscillations.

As shown in Figure S6, the resulting fit yields a characteristic domain spacing (d) of 4.2 nm and a structural correlation length (ξ) of 9.7 nm. The excellent goodness-of-fit (R^2 = 0.9913, RMSE = 0.0186) confirms that the mesophase geometry is highly consistent with the Teubner-Strey model for bicontinuous microemulsions.

1.2. Glycerol

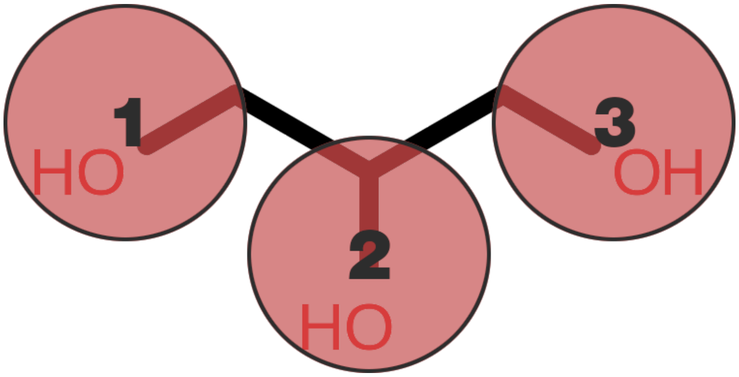

Figure S7: Mapping scheme for glycerol. Numbers on the beads correspond to Table S4.

Table S4: Topology of Martini 3 glycerol.

Bead definitions			
**Number**	**Bead type**	**Charge**	**Mass**
1	TP1d	0.00	31
**2**	TP1dr	0.00	30
**3**	TP1d	0.00	31

Bonds				
i	***j***	**Function**	**Length**	**Force c.**
**1**	2	1	0.255	2000
**2**	3	1	0.255	2000
**3**	1	1	0.310	1000

A note on the angle: From AA simulations the 1-2-3 angle should be ~70 degrees. Both angle types 1 and 2 struggle keeping this with low force constants, and therefore it is both computationally cheaper as well as more stable to use a bond between 3 and 1.

Table S5: Measured thermodynamic properties for glycerol.

Property	Martini 3	Predicted	Experimental	Ref.
**Log P**	-2.00	-1.93 (ALOGPS)	-1.76	4

Table S6: Solvent accessible surface area (SASA). AA value is derived using Rowland's atomic radii. Positive difference indicates the Martini model to be larger than the atomistic model, and vice versa.

Martini 3 (nm^2)	All atom (nm^2)	Difference (%)
2.959 ± 0.08	3.077 ± 0.03	-3.83

In order to at least partially quantify the extent of the so-called 'Martini hangover' one would obtain from using this model, we evaluated the behaviour of glycerol over a range of temperatures and compared the results to those from Ahmadi *et al.*[5] For a 91:9 glycerol:water system, expressed as linear functions, the densities are -0.566 * T + 1400.85 for experiment (R^2 = 0.999) and -1.301 * T + 1690.28 (R^2 = 0.945) for Martini 3 glycerol. The largest deviation from experiment over the whole range is 7%, with average being 5%. The discrepancy decreases with increasing temperature and is approximately 2.6% at 350 K.

2. Micelle morphologies

Table S7: Summary of CTAB aggregate morphological parameters at 298 K. R_1, R_2, and R_3 are the time-averaged square roots of the eigenvalues of the mass-weighted gyration tensor; R_g is the total radius of gyration; and K^2 is the relative shape anisotropy. Shape metrics are reported as mean (SD) over the final 50 ns. The 95% confidence interval for K^2 was obtained by block bootstrap using ten contiguous 5 ns blocks. Average box lengths are reported as mean (SD).

**Glycerol:Urea**	R_1	R_2	R_3	R_g	K^2	K^2 *95% bootstrap CI*	**Avg. box length**
**-**	**Å**				**-**	**-**	**Å**
1:9	19.43 (0.39)	18.41 (0.36)	17.08 (0.47)	31.76 (0.10)	0.006 (0.004)	[0.0047, 0.0084]	121.9 (0.005)
2:8	19.56 (0.52)	18.24 (0.32)	17.12 (0.37)	31.77 (0.10)	0.007 (0.005)	[0.0051, 0.0089]	122.2 (0.005)
3:7	19.61 (0.47)	18.22 (0.39)	17.05 (0.39)	31.74 (0.09)	0.008 (0.004)	[0.006, 0.0091]	122.5 (0.006)
4:6	19.34 (0.41)	18.16 (0.26)	17.31 (0.35)	31.68 (0.08)	0.005 (0.003)	[0.0039, 0.0059]	122.8 (0.005)
5:5	19.32 (0.42)	18.27 (0.29)	17.20 (0.37)	31.69 (0.08)	0.005 (0.003)	[0.0038, 0.0066]	121.6 (0.002)
6:4	19.33 (0.45)	18.15 (0.28)	17.26 (0.30)	31.64 (0.08)	0.005 (0.004)	[0.0034, 0.0058]	121.7 (0.002)
7:3	19.23 (0.44)	18.15 (0.31)	17.29 (0.33)	31.60 (0.08)	0.005 (0.003)	[0.0034, 0.0059]	121.9 (0.003)
8:2	19.16 (0.32)	18.17 (0.27)	17.26 (0.31)	31.55 (0.07)	0.004 (0.002)	[0.0037, 0.0046]	122.1 (0.002)
9:1	18.98 (0.31)	18.14 (0.24)	17.36 (0.26)	31.48 (0.06)	0.003 (0.002)	[0.0027, 0.0035]	122.3 (0.003)
Glyceline	19.48 (0.36)	18.33 (0.33)	17.24 (0.33)	31.83 (0.07)	0.006 (0.003)	[0.0049, 0.0062]	124.9 (0.003)

Table S8: Summary of CTAB aggregate morphological parameters at 350 K. R_1, R_2, and R_3 are the time-averaged square roots of the eigenvalues of the mass-weighted gyration tensor; R_g is the total radius of gyration; and K^2 is the relative shape anisotropy. Shape metrics are reported as mean (SD) over the final 100 ns. The 95% confidence interval for K^2 was obtained by block bootstrap using ten contiguous 10 ns blocks. Average box lengths are reported as mean (SD).

**Glycerol:Urea**	R_1	R_2	R_3	R_g	K^2	K^2 *95% bootstrap CI*	**Avg. box length**
**-**	**Å**				**-**	**-**	**Å**
9:1	16.08 (0.55)	14.58 (0.38)	13.24 (0.42)	25.44 (0.13)	0.014 (0.008)	[0.0126, 0.0162]	176.3 (0.004)
5:5	20.65 (0.96)	17.53 (0.84)	9.13 (0.19)	28.61 (0.31)	0.141 (0.024)	[0.1334, 0.1501]	172.7 (0.003)
3:7	20.46 (0.83)	17.94 (0.72)	9.07 (0.18)	28.70 (0.25)	0.136 (0.017)	[0.1308, 0.1428]	173.6 (0.004)

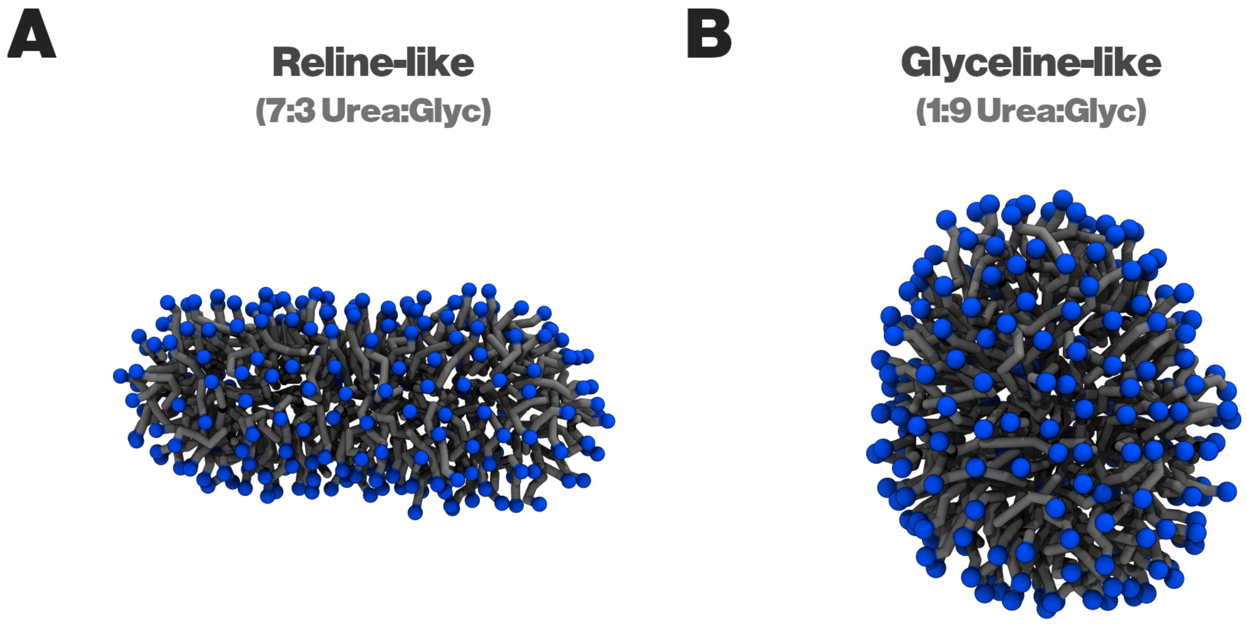

Figure S8: Representative simulation snapshots of the composition-dependent morphological transition of CTAB micelles at 350 K. (A) An anisotropic, non-spherical aggregate in the urea-rich Reline-like environment. (B) A near-globular aggregate maintained in the glycerol-rich Glyceline-like environment.

3. Radial Distribution Functions

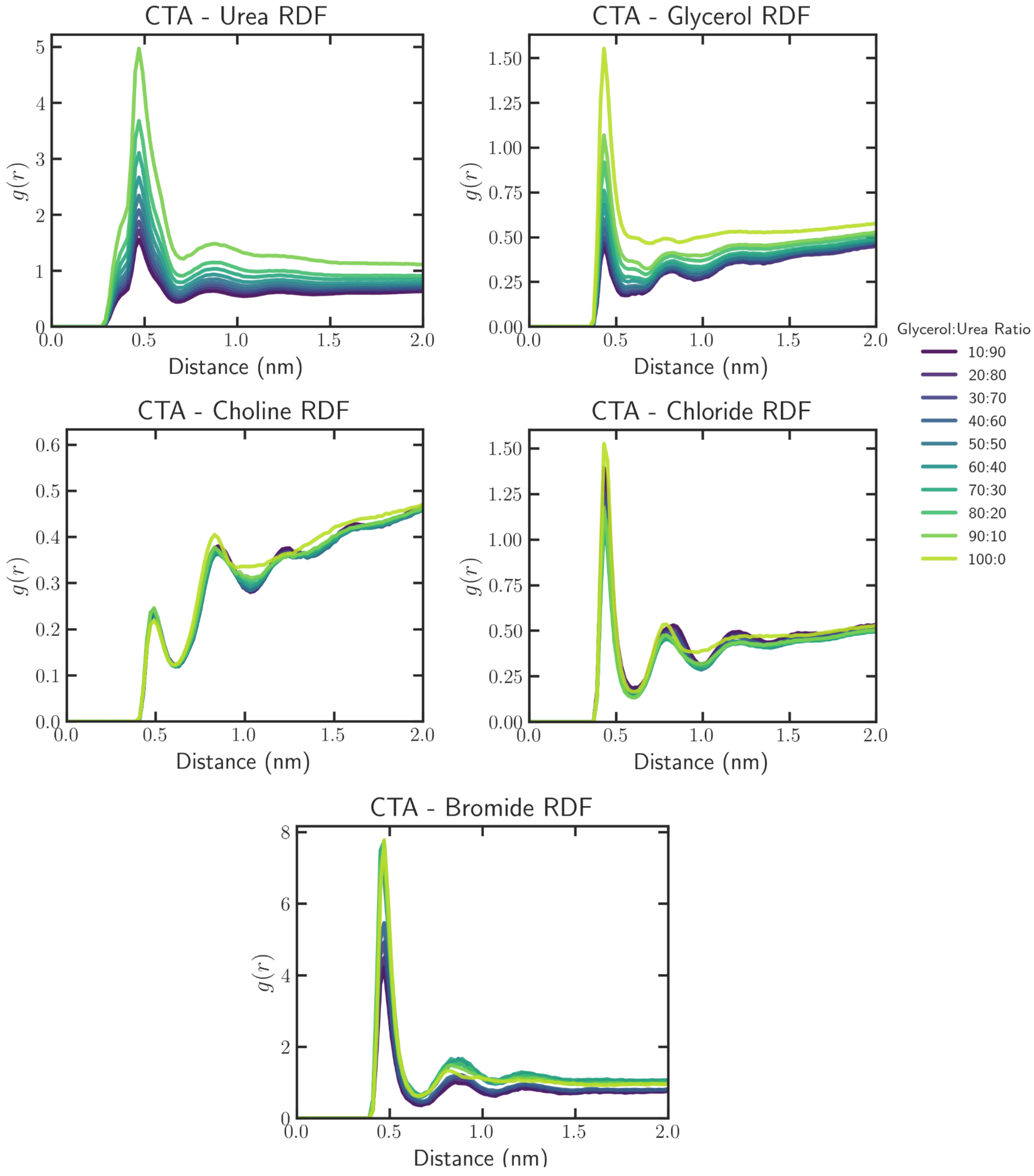

Figure S9: CTA-cation - solvent and ion interactions over a range of compositions at 298 K.

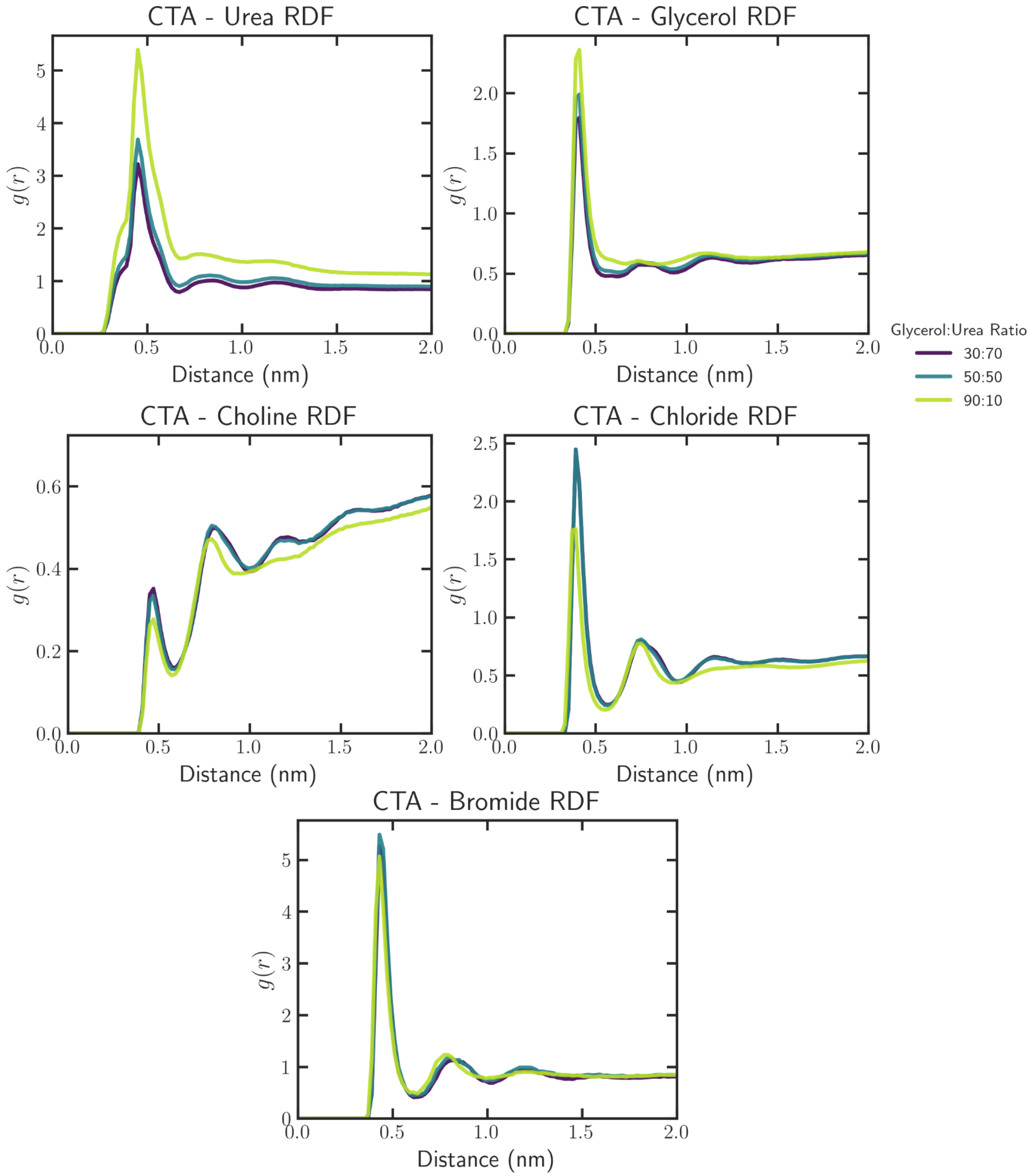

Figure S10: CTA-cation - solvent and ion interactions over a selected range of compositions, at a significantly elevated temperature (350 K). Profiles are shown for the 30:70, 50:50, and 90:10 Glycerol:Urea mixtures.

4. Minimum Distance Distribution Function Density Maps

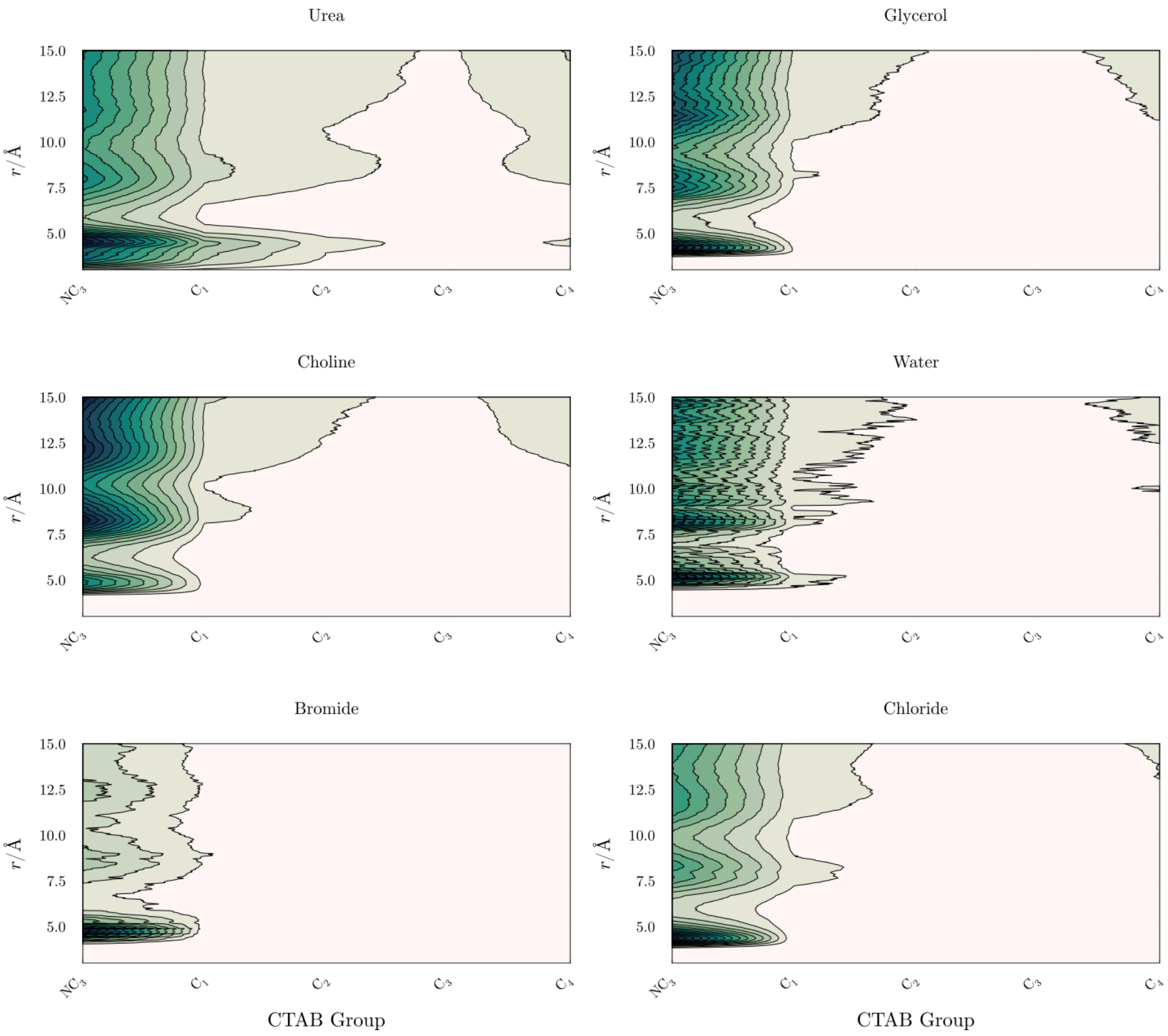

Figure S11: CTA-cation - solvent and ion MDDF density maps for 10% glycerol.

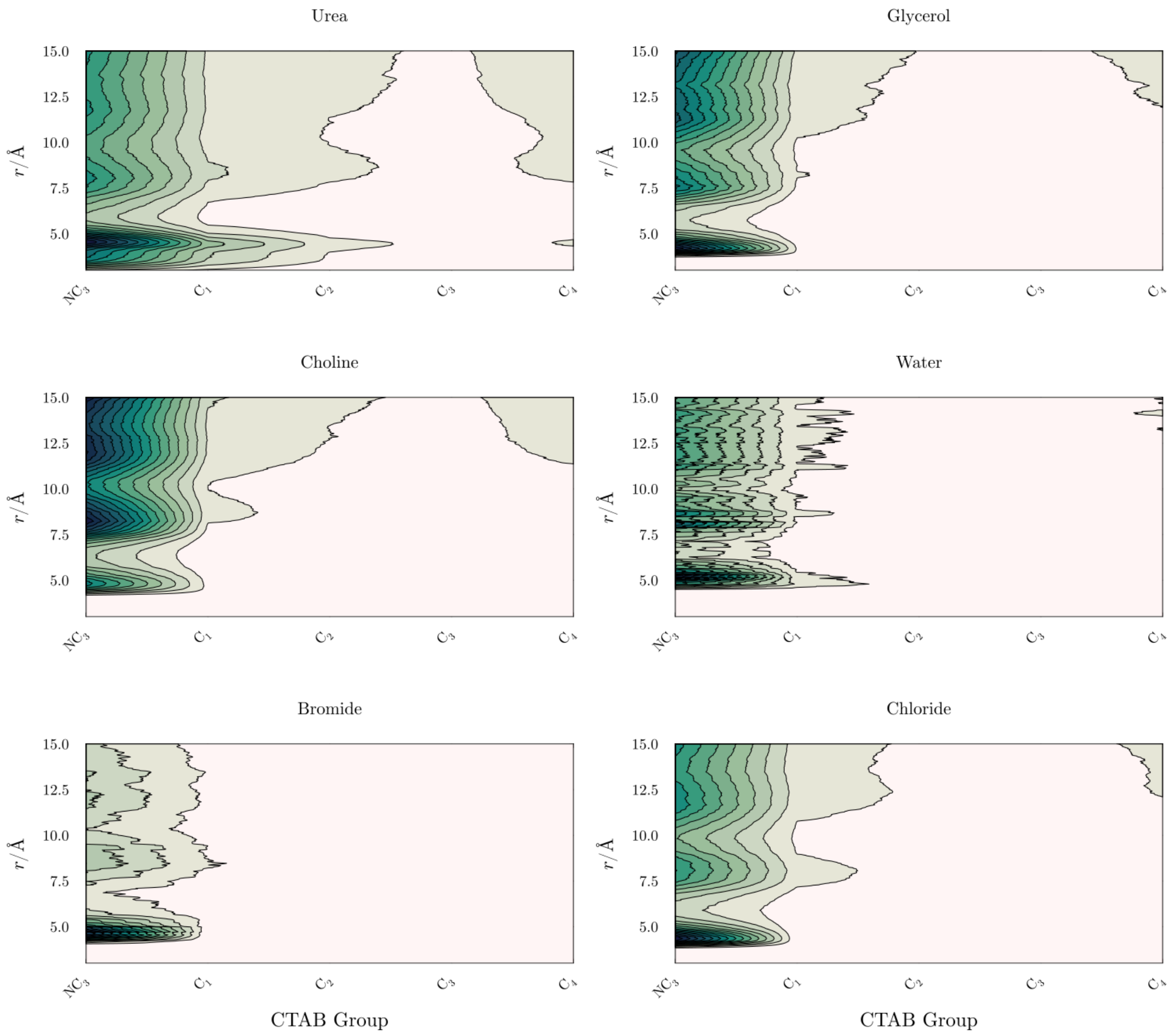

Figure S12: CTA-cation - solvent and ion MDDF density maps for 20% glycerol.

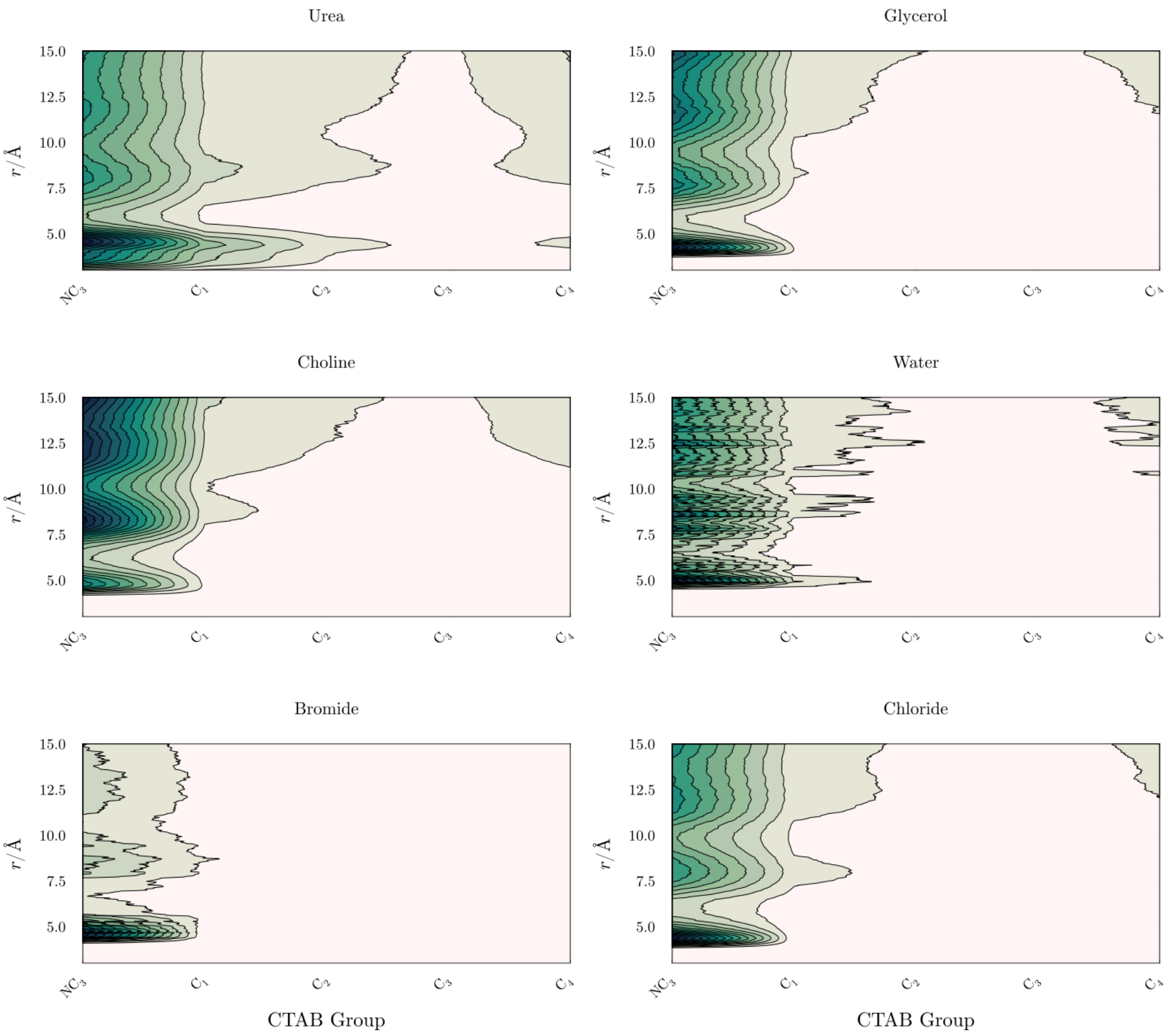

Figure S13: CTA-cation - solvent and ion MDDF density maps for 30% glycerol.

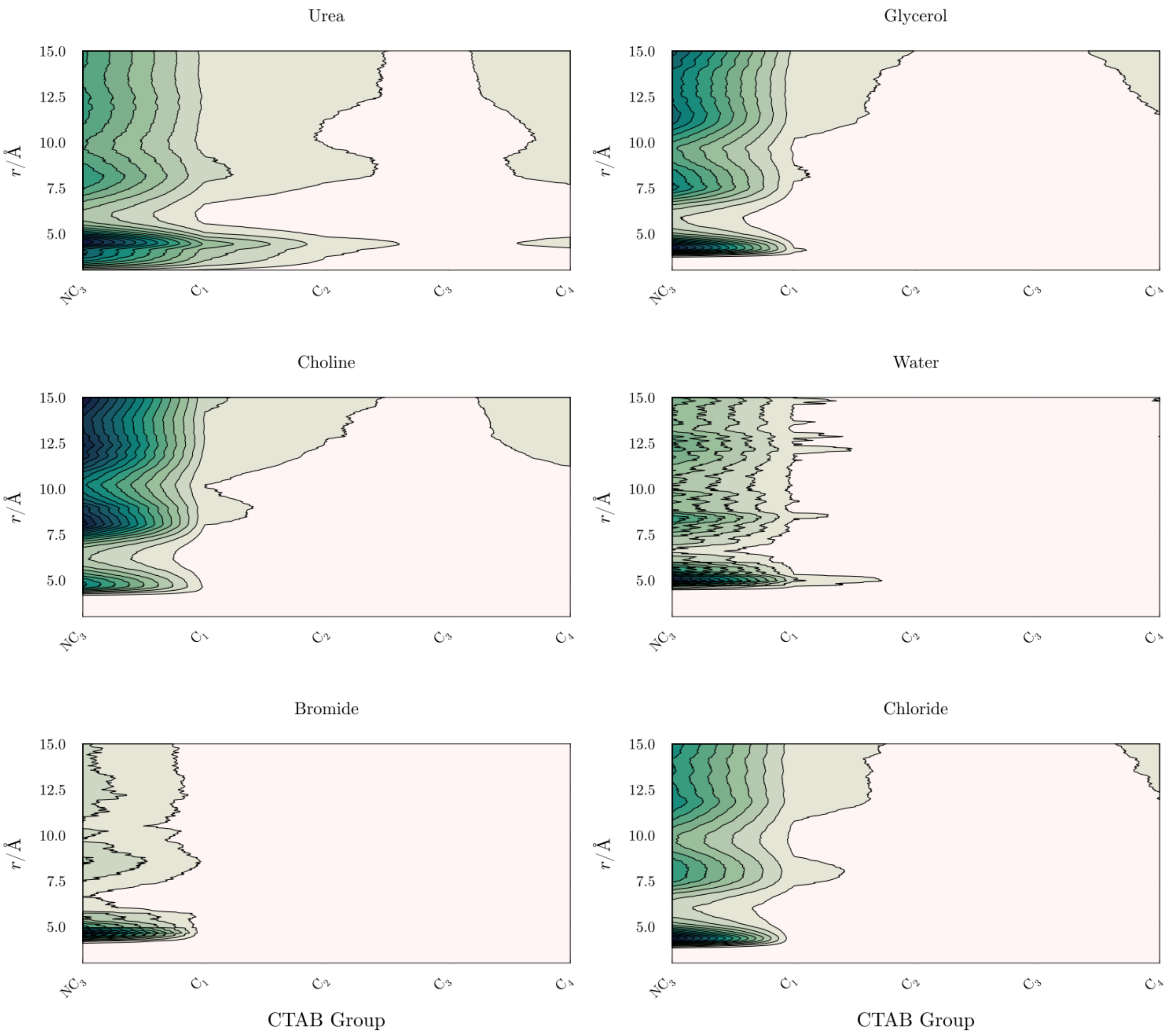

Figure S14: CTA-cation - solvent and ion MDDF density maps for 40% glycerol.

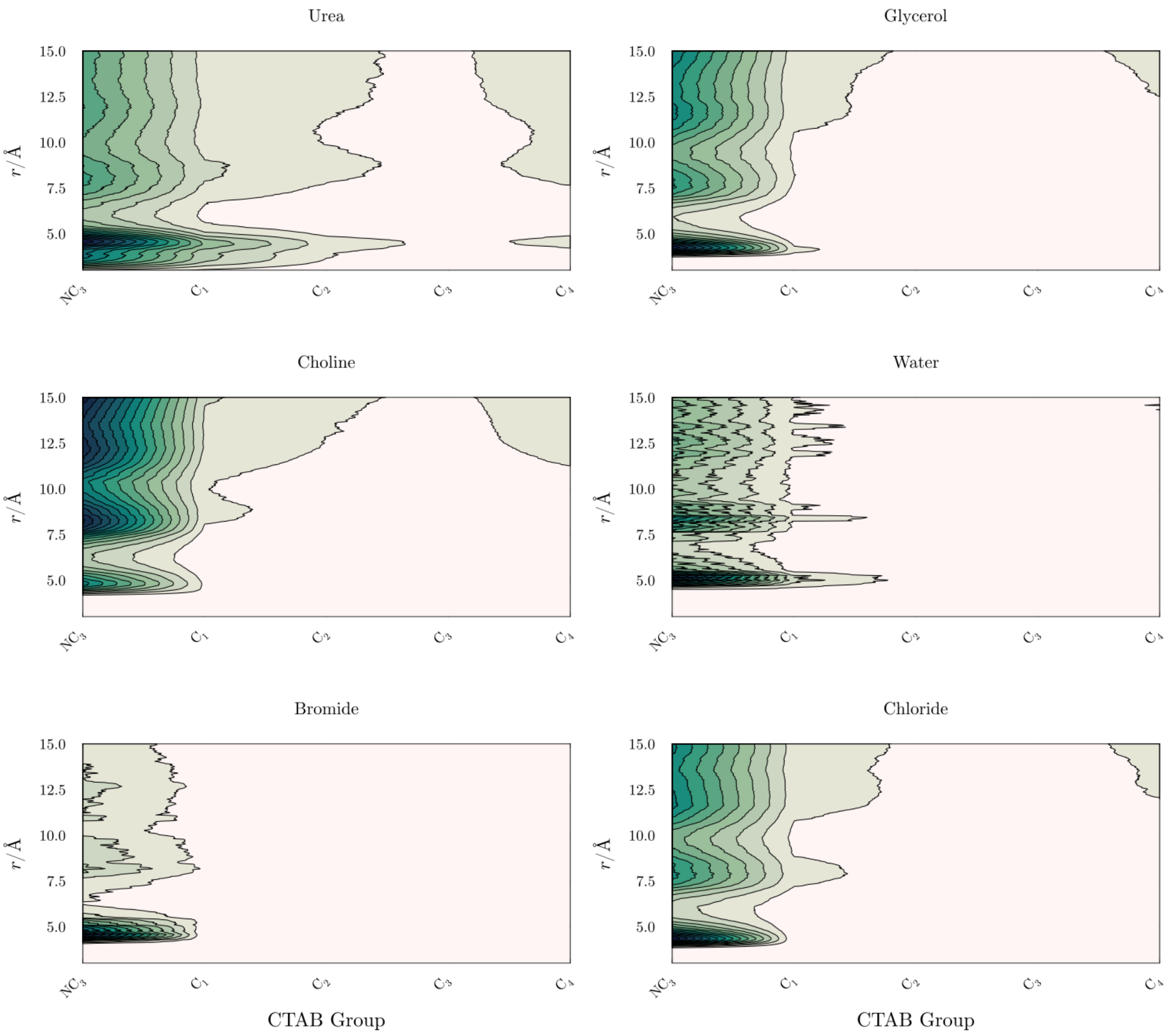

Figure S15: CTA-cation - solvent and ion MDDF density maps for 50% glycerol.

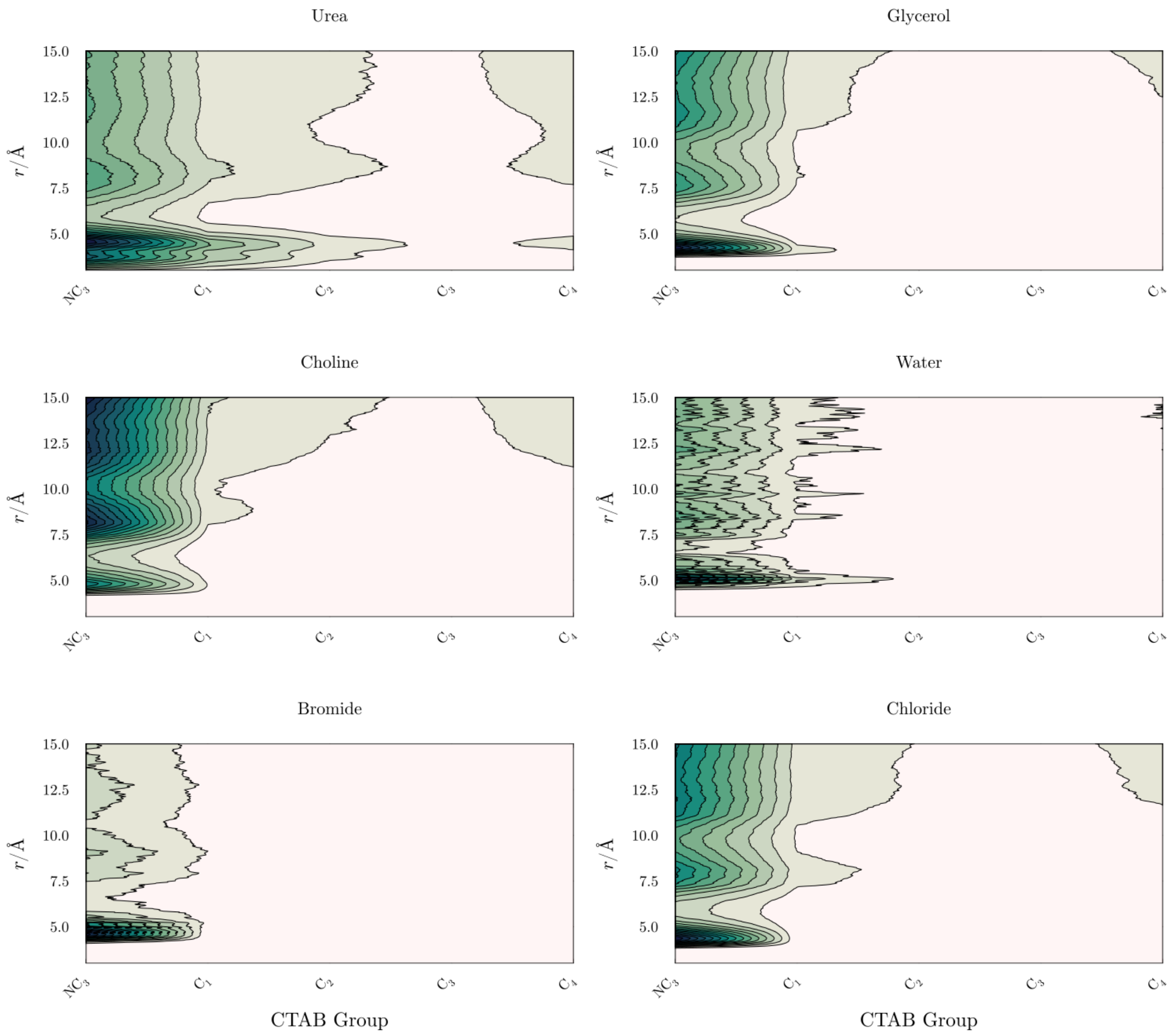

Figure S16: CTA-cation - solvent and ion MDDF density maps for 60% glycerol.

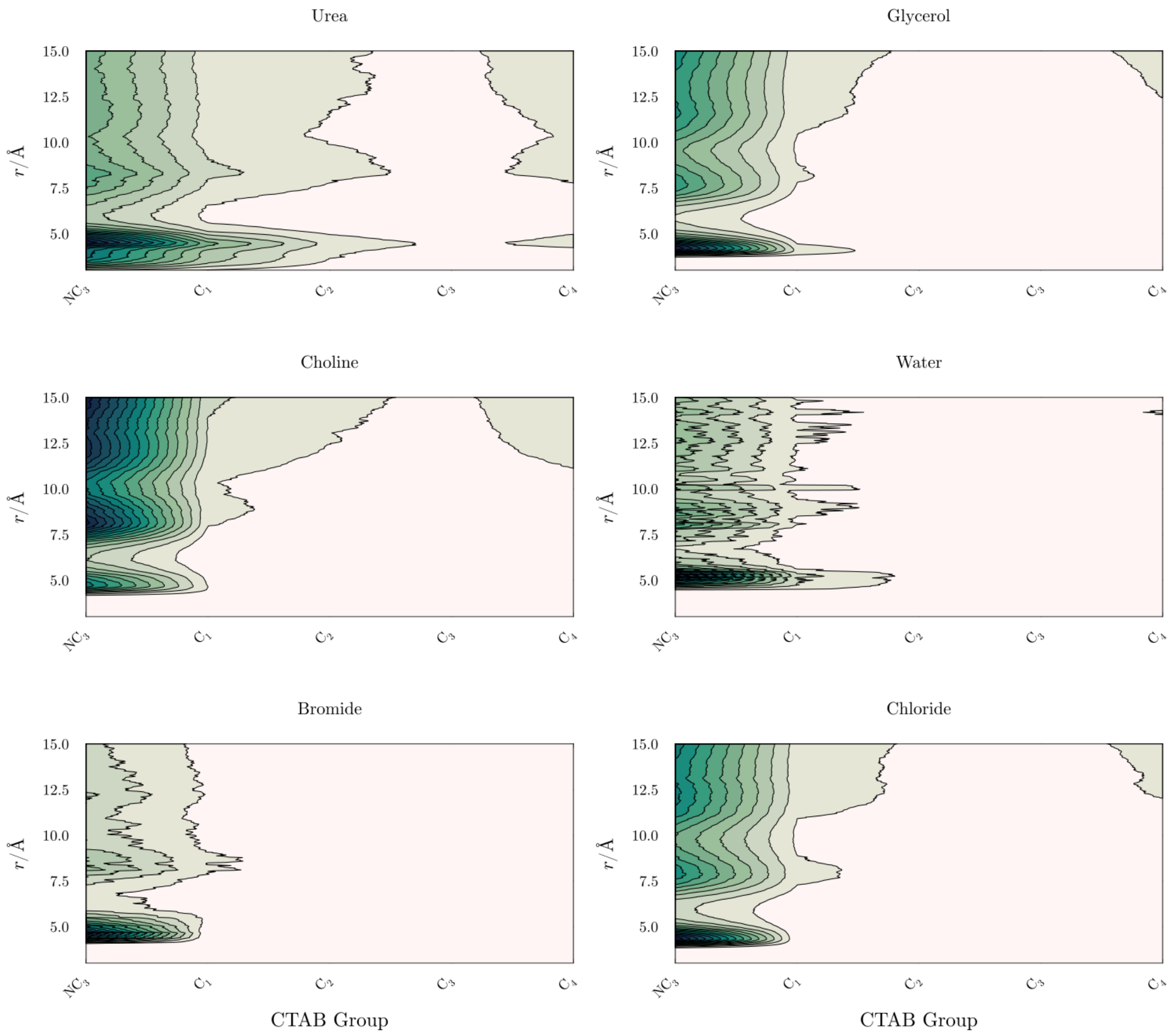

Figure S17: CTA-cation - solvent and ion MDDF density maps for 70% glycerol.

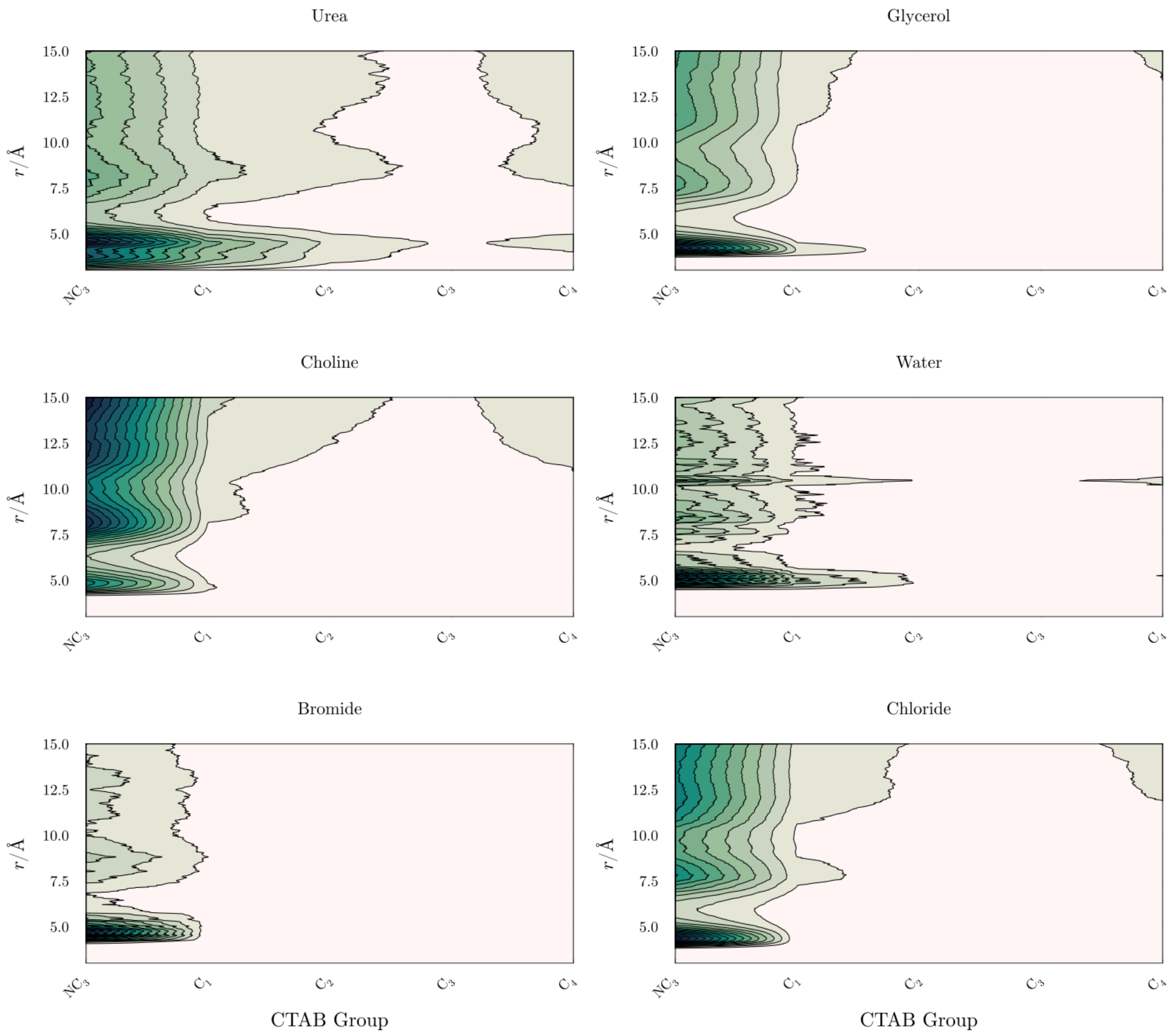

Figure S18: CTA-cation - solvent and ion MDDF density maps for 80% glycerol.

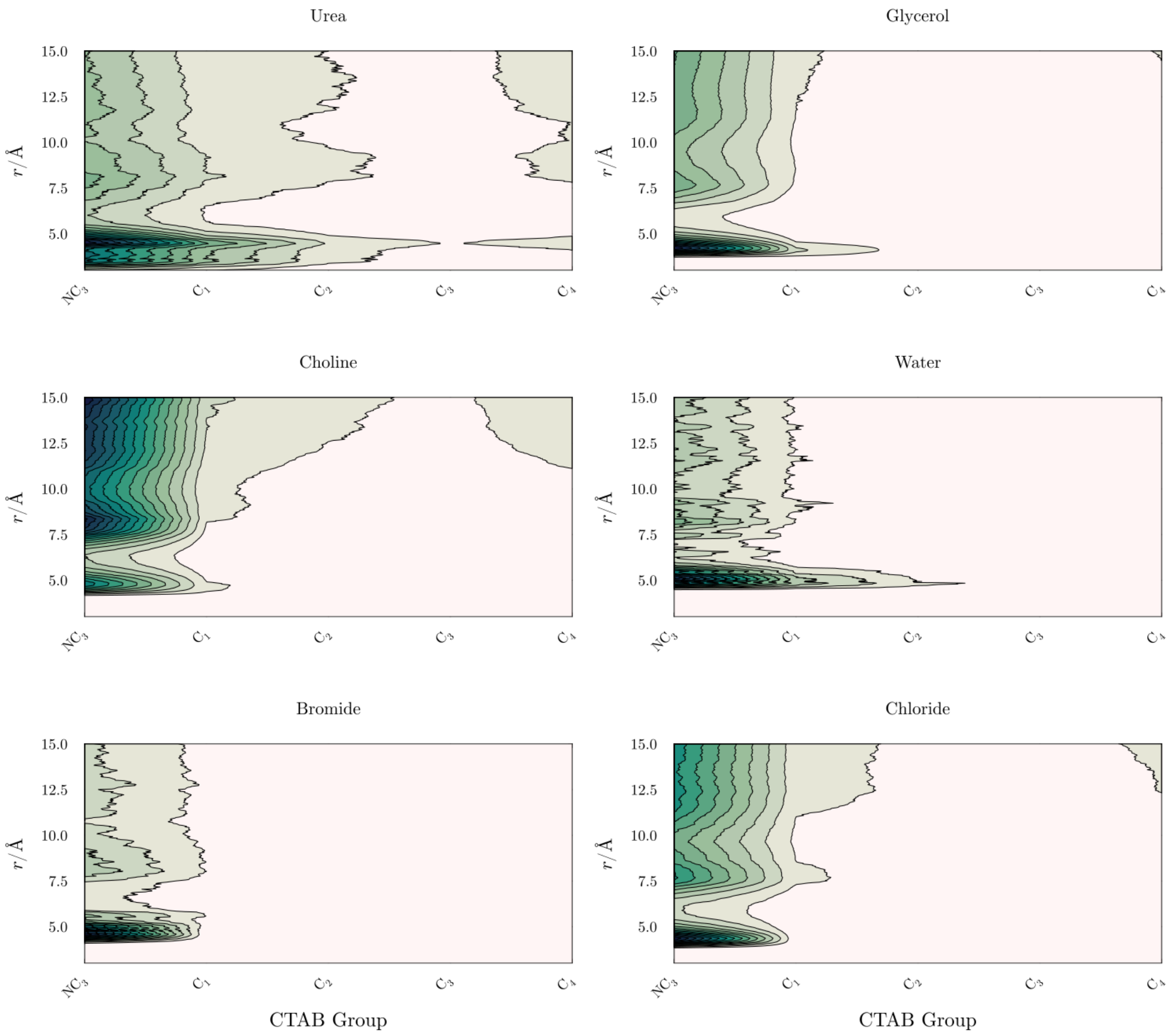

Figure S19: CTA-cation - solvent and ion MDDF density maps for 90% glycerol.

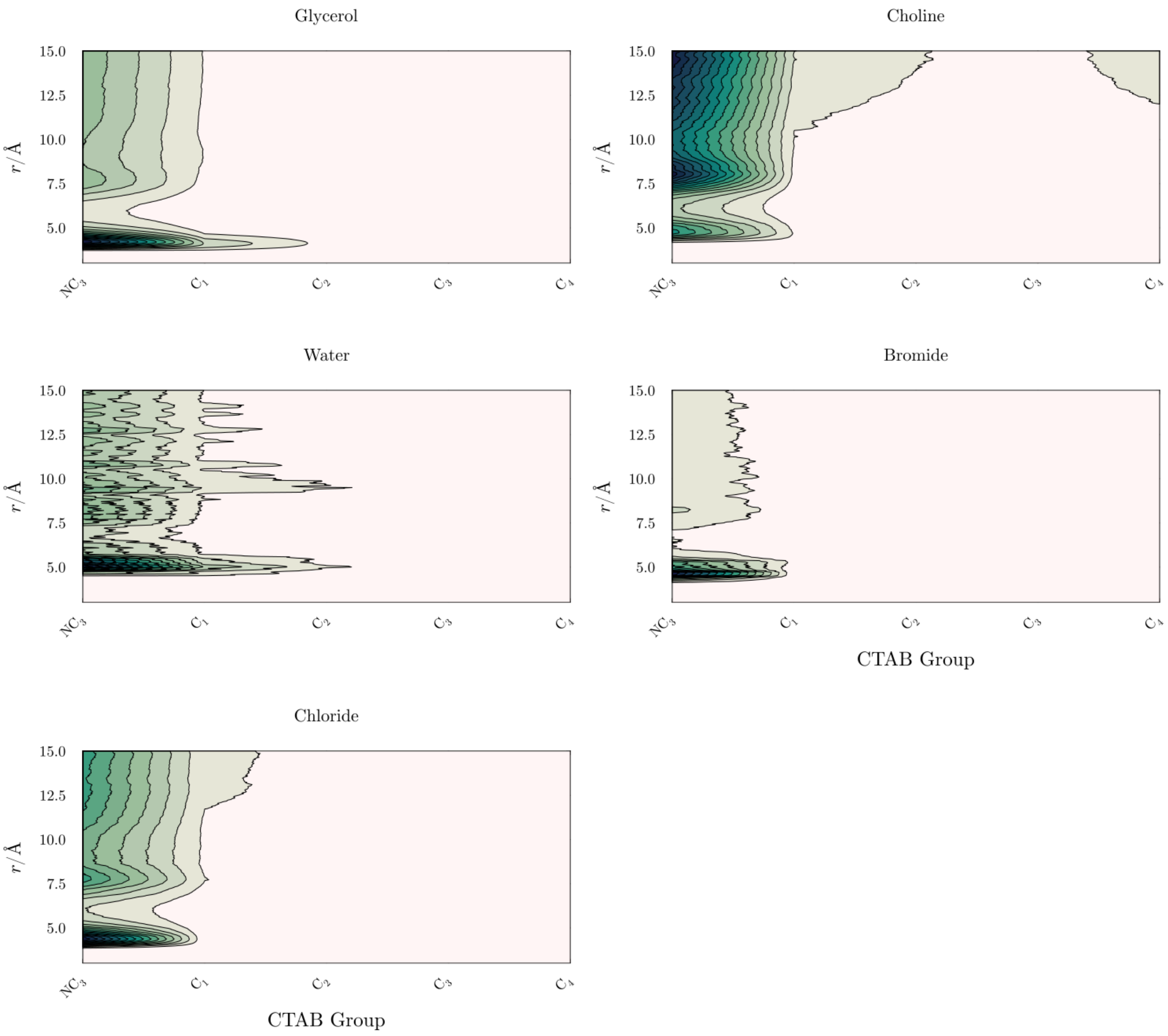

Figure S20: CTA-cation - solvent and ion MDDF density maps for glyceline (no urea in the mixture).

References

1. Majhi, P.; Moulik, S. Energetics of Micellization: Reassessment by a High-Sensitivity Titration Microcalorimeter. *Langmuir,* 1998, 14, 15, 3986–3990
2. Rowland, R. S.; Taylor, R. Intermolecular Nonbonded Contact Distances in Organic Crystal Structures: Comparison with Distances Expected from van der Waals Radii. *J. Phys. Chem.* 1996, 100, 7384– 7391.

3. Varade, D.; Aramaki, K.; Stubenrauch, C. Phase diagrams of water–alkyltrimethylammonium bromide systems. *Colloids and Surfaces A: Physicochemical and Engineering Aspects*. 2008, 315, 1–3, 205-209.
4. Hansch, C.; Dunn, W. J. Linear Relationships between Lipophilic Character and Biological Activity of Drugs. *J. Pharm. Sci*. **1972**, 61, 1– 19.
5. Ahmadi, P.; Chapoy, A.; Burgass, R. An investigation on the thermophysical properties of glycerol.*The Journal of Chemical Thermodynamics.* 2023, 178, 106975.
